# Severely malnourished children with a low weight-for-height have similar mortality to those with a low mid-upper-arm-circumference: II. Systematic literature review and meta-analysis

**DOI:** 10.1186/s12937-018-0383-5

**Published:** 2018-09-15

**Authors:** Emmanuel Grellety, Michael H. Golden

**Affiliations:** 1 Research Center Health Policy and Systems - International Health, School of Public Health, Université Libre de Bruxelles, Bruxelles, Belgium; 2 Department of Medicine and Therapeutics, University of Aberdeen, Aberdeen, Scotland, UK

**Keywords:** Nutrition, Acute malnutrition, Severe acute malnutrition, SAM, Mid-upper-arm circumference, MUAC, Weight-for-height, WHZ, Mortality, Case fatality rate, Wasting, Oedema, Kwashiorkor, Diagnosis, Meta-analysis, Systematic review, Simpson’s paradox, Child, Human

## Abstract

**Background:**

The WHO recommended criteria for diagnosis of sever acute malnutrition (SAM) are weight-for-height/length Z-score (WHZ) of <− 3Z of the WHO_2006_ standards, a mid-upper-arm circumference (MUAC) of < 115 mm, nutritional oedema or any combination of these parameters. A move to eliminate WHZ as a diagnostic criterion has been made on the assertion that children with a low WHZ are healthy, that MUAC is a “superior” prognostic indicator of mortality and that adding WHZ to the assessment does not improve the prediction of death. Our objective was to examine the literature comparing the risk of death of SAM children admitted by WHZ or MUAC criteria.

**Methods:**

We conducted a systematic search for reports which examined the relationship of WHZ and MUAC to mortality for children less than 60 months. The WHZ, MUAC, outcome and programmatic variables were abstracted from the reports and examined. Individual study’s case fatality rates were compared by chi-squared analysis and random effects meta-analyses for combined data.

**Results:**

Twenty-one datasets were reviewed. All the patient studies had an ascertainment bias. Most were inadequate because they had insufficient deaths, used obsolete standards, combined oedematous and non-oedematous subjects, did not report the proportion of children with both deficits or the deaths occurred remotely after anthropometry. The meta-analyses showed that the mortality risks for children who have SAM by MUAC < 115 mm only and those with SAM by WHZ < −3Z only are not different.

**Conclusions:**

As the diagnostic criteria identify different children, this analysis does not support the abandonment of WHZ as an important independent diagnostic criterion for the diagnosis of SAM. Failure to identify such children will result in their being denied treatment and unnecessary deaths from SAM.

**Electronic supplementary material:**

The online version of this article (10.1186/s12937-018-0383-5) contains supplementary material, which is available to authorized users.

## Background

Severe acute malnutrition (SAM) affects at least 19 million children at any one time [1]. Identification of all these children and admission to treatment programs is a public health priority. The World Health Organisation (WHO) has established three independent criteria for the diagnosis of children with SAM. These are a weight-for-height/weight-for-length Z-score (WHZ) of <−3Z or an absolute mid-upper-arm circumference (MUAC) of < 115 mm to assess marasmus, and bilateral nutritional oedema to include kwashiorkor and marasmic-kwashiorkor when both oedema and an anthropometric deficit are present.

Because of its simplicity, ease of use and relative cheapness as a screening tool MUAC has been readily taken up to identify and treat children with SAM in the community and elsewhere [2]. Thus, MUAC has been widely adopted by many agencies and several governments as the only anthropometric criterion for SAM; they consider WHZ cumbersome and difficult to assess. However, although the prevalence of SAM is about the same in representative community nutritional surveys, different children are identified by the two anthropometric criteria with a considerable discordance in individual countries [3–20]. Data from representative community samples of children from 47 countries [21] show that the overall overlap for anthropometric SAM (i.e. children fulfilling both the WHZ and MUAC criteria) was only 16.5%. About 45% of the children fulfil the WHZ definition for SAM but not the MUAC criterion. Where a MUAC only policy has been adopted children fulfilling WHO’s WHZ criterion, but not the MUAC criterion, for SAM remain un-identified and un-treated.

Because the two diagnostic parameters select different children we proposed that both MUAC and WHZ have to be retained and continue to be used routinely to identify all those children who need to be treated for SAM and methods found to more easily identify those children with a low WHZ in the community [21]. These proposals led to forceful criticism from Briend et al. [22] who maintain that only MUAC should be used to identify severely malnourished children and that the use of WHZ can be safely dispensed with. This proposal appears to have widespread approval [23–28].

Briend et al. [22, 26] contend that children with a low WHZ do not need to be identified or treated on the grounds that, 1) MUAC has been repeatedly shown to be a “superior” diagnostic parameter to predict subsequent death of SAM children, 2) that children with a low WHZ are healthy and thus do not need treatment; 3) that they only have a low WHZ because their legs are relatively long, 4) that the two anthropometric criteria are proxies for each other, 5) that when children satisfy both criteria their mortality rate is not additive, but that MUAC mortality is universally higher than that with WHZ [22, 26, 29–31] and 6) that addition of WHZ to MUAC does not increase the prognostic sensitivity or specificity of future death prediction and is therefore redundant [31]. These repeated assertions have consequently led to failure to identify children with a WHZ < −3Z, but a normal MUAC, in many programs and officially by some governments who have abandoned the use of WHZ altogether. What started as a simple screening tool for the community identification and as an alternative to WHZ has changed into the primary tool to be used to diagnose SAM with advocacy to extend the suppression of WHZ assessment universally [22, 29, 30, 32].

This series of papers addresses the veracity of these assertions. As we consider whether all the criteria for SAM should be routinely used, or whether WHZ assessment can be omitted safely, the mortality risk of children who would be excluded from diagnosis and treatment as a result of such a policy must be the focus of consideration. As the fate of millions of SAM children is at stake, such a change of policy must be based upon unequivocal robust evidence. In paper I [33] we present empirical data to show that children under treatment for SAM have a higher mortality when admitted with the WHZ criterion, without fulfilling the MUAC criterion, than with the MUAC criterion without fulfilling the WHZ criterion. In paper III [34] we examine the influence of the relative case-load with each criterion on the potential avoidable deaths that would occur in excluded children.

The objective of this paper is to examine the evidence in the published literature that compares the risk of death of severely malnourished children who satisfy the WHZ with those that satisfy the MUAC criteria and to assess whether those that fulfil the WHZ criterion only can be safely omitted from treatment programs.

## Methods

We used the Preferred Reporting Items for Systematic Reviews and Meta-Analysis (PRISMA) statement to guide our review [35].

### Papers included in the analysis

In order to exhaustively include as much evidence as possible pertaining to mortality risk of SAM children by the two anthropometric criteria, all studies were eligible for inclusion if they contained the numbers of children, less than 60 months of age, that were alive and dead when they exited the study, and who had SAM by any WHZ and any MUAC criteria, in any language and at any time.

**Fig. 1:**
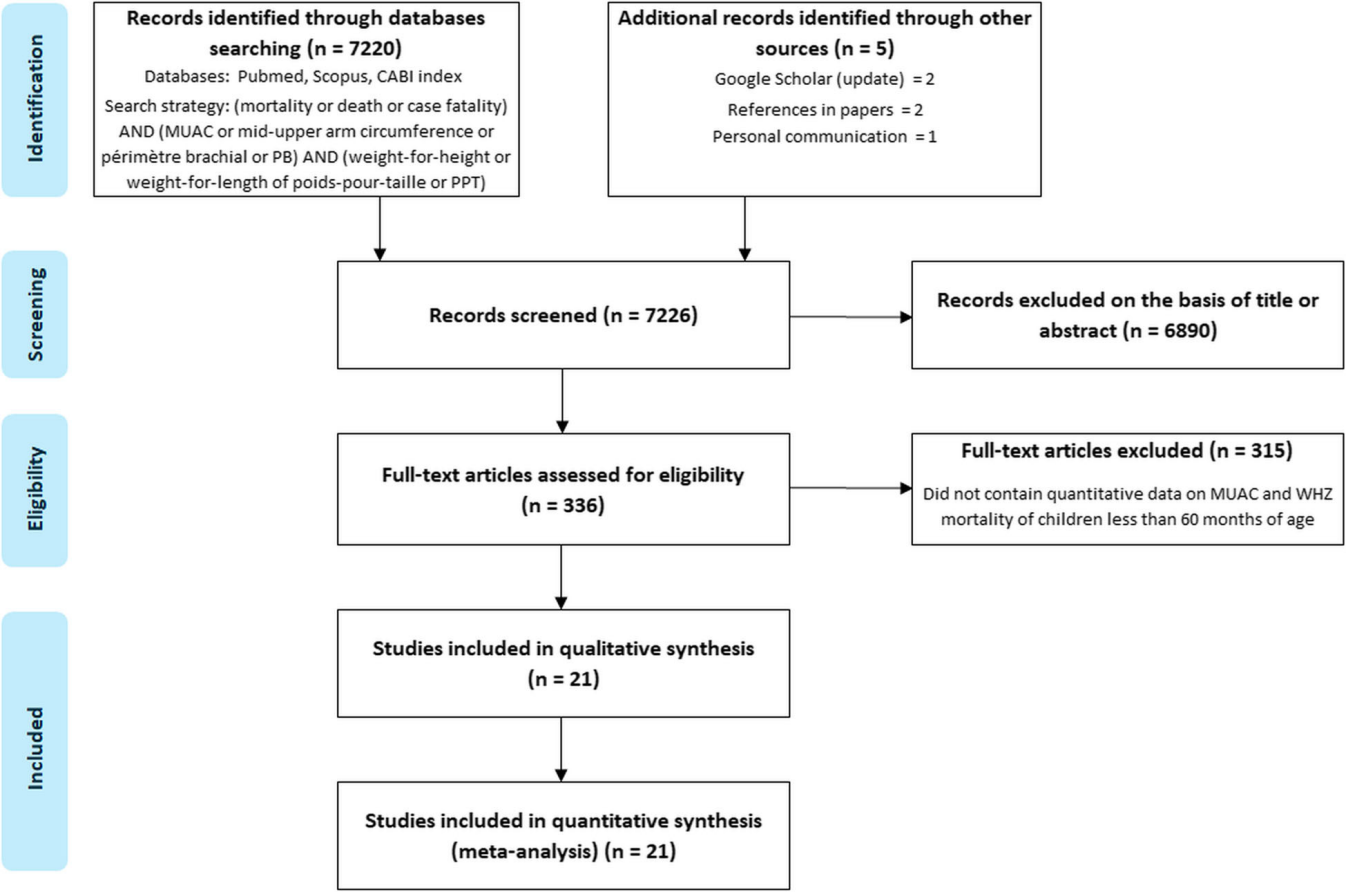
Flow of study selection

In February 2017 we searched Pubmed, Scopus and CABI- index for papers of relevance using the search terms: (Mortality or Death or Case Fatality) and (MUAC or mid-upper-arm circumference or arm-circumference or Perimeter Brachial) and (WHZ or weight-for-height or weight-for-length or poids pour taille or PPT). Google-Scholar was also regularly consulted, particularly for papers indexed after our initial search. The search strategy is shown in Fig. 1. Papers that did not provide the numbers of children with MUAC, WHZ and outcome were not considered. Papers relating to children with primary disease unrelated to nutrition were not considered (e.g. congenital heart disease, chronic renal failure, malignancies, cystic fibrosis, etc.). Where possible the original theses, reports and supplementary information from which the paper was written were obtained as these usually gave fuller information. The data presented in our companion paper [33] were not included. One conference presentation [36] was included in the analysis. From the final 20 reports (21 datasets) containing relevant information the following data were abstracted: authors and reference: date: place: type and purpose of study: study setting, in-patient facility (IPF), out-patient treatment program (OTP) or Community cohort: the length of time the children were observed: the standards used for diagnosis of SAM by MUAC and WHZ: the age range of the children included: whether oedematous children were included or excluded: the percent of overlap between the two parameters (i.e. those fulfilling both WHZ and MUAC criteria for SAM / total SAM): the percent of missing data: the default rate: the total number of children and the number that died in the WHZ and MUAC categories. Were possible the number of children fulfilling both diagnostic criteria was obtained and these reports then formed a separate category. It was not possible to estimate the proportions of overlap for those reports where this information was not given.

### Statistical analysis

The original authors analysed their data in a number of ways and some did not present results that directly compared the risk of death of SAM children diagnosed by MUAC, WHZ or both. Most analysed all-cause mortality in the whole childhood community or patient population, including children who did not have SAM by any definition. The area-under a ROC curve, logistic regression, life table, etc., including all available children, were then used in the analysis. For the present analysis the subjects that did not have SAM were not considered; this was because the objective was not to compare SAM children with non-SAM children, but to compare the relative mortality risks of children with SAM who fulfilled either the MUAC or the WHZ criterion. Therefore the original analyses were not considered. The actual numbers of children who were below the cut-off points used in the paper to define SAM who survived and those who died were used to re-analyse the data. We directly compared the case fatality rates (CFR) and relative risk (RR) of death of children with SAM by MUAC with SAM by WHZ.

There were 10 datasets where children with SAM could be divided into the following groups:

SAM by MUAC but not by WHZ criteria – designated as “S-muac”

SAM by WHZ but not by MUAC criteria – designated as “S-whz”

SAM by both MUAC and WHZ criteria – designated as “S-both”

There were 11 datasets where children with SAM by both criteria were not separately reported from those children with SAM by one or the other criterion (i.e. S-both could not be determined). In these reports children with SAM by MUAC may, or may not, also have SAM by WHZ; they are designated as “All-muac”. Similarly, those with SAM by WHZ may, or may not, also have SAM by MUAC; they are designated as “All-whz”. These papers were potentially in error because of mathematical coupling (see companion paper I [33]) and are examined as a separate group. Mathematical coupling occurs where “*one variable directly or indirectly contains the whole or part of another, and the two variables are analysed using standard statistical techniques*” [37, 38]. Thus, S-muac, S-whz and S-both data are mutually exclusive, but All-muac and All-whz are not mutually exclusive. There were also papers where oedematous children were incorporated into the analysis, usually with an unknown proportion of oedematous children in each group; this is known to be a major confounder [33]. Thus, where oedematous children were included in the dataset, so that marasmus could not be differentiated from marasmic-kwashiorkor the reports were analysed in a sub-category.

The data from two studies were each reported using different standards (study from Senegal in datasets 18 & 19 [39, 40] and from Democratic Republic of Congo in datasets 20 & 21 [40, 41]). Each of the duplicate datasets were included in an initial analysis by subgroup comparing the effect of using different diagnostic cut-off points to analyse the data and demonstrate the effect of using different references on the derived outcomes. The duplicated data (18 to 21) were not used in the other analyses as report 7 had incorporated the same two datasets, combined with a study from Nepal, into their analysis. As report 7 used WHO criteria, separated children with both criteria (S-both) and as far as possible excluded oedematous children the data in this report was considered to be most reliable.

Individual datasets with S-muac vs, S-whz and All-muac vs All-whz were analysed by 2 × 2 chi-squared. Where there were fewer than 5 children in any expected category the analysis was by Fisher’s exact test.

### Meta-analyses

The data abstracted from the individual papers were examined by meta-analyses using MetaXL version 5.3 software [42]; the random effects model with relative risk (RR) output divided by subgroups, was used to compare the effects of potential bias. The quality effects model was used for the final analysis where the studies are grouped by whether S-both data were available or not [43]. Tests for heterogeneity were generated for each analysis by two methods (funnel plots and Doi plot). In the funnel plot, the ORs were plotted against the standard error, while in Doi plots, the ORs were plotted against z-score. To examine the potential effects of bias because of the differences in the study type, standards and inclusion criteria, the meta-analyses were repeated dividing the studies into the following sub-categories: 1) to test the biasing effect of oedema inclusion, S-muac v S-whz with and without oedematous case inclusion (i.e. omitting the 11 studies with only All-muac and All-whz information): 2) to test the effects of using different definitions of SAM, the datasets were divided by the standards and cut-off points for diagnosis: 3) to examine ascertainment and treatment bias, the studies were grouped by type of study (community cohort, in-patients treated for SAM, out-patients treated for SAM): and, 4) the difference between those that excluded children with S-both and those that incorporated them into their dataset. The assessment of study quality and potential for bias was assessed, using the criteria in table Additional file 1: Table S1. Confidence intervals are 95% for all reports and a probability of 0.05 is considered significant.

### Effect of including or excluding “S-both” in the analyses

In order to examine the effects of including children suffering from both deficits (i.e. WHZ < −3Z and MUAC < 115 mm) in the comparison of mortality related to WHZ and MUAC, we calculated both the CFRs of S-whz and S-muac and for the same cohorts of SAM children when S-both was added to S-whz and S-muac to give the corresponding All-whz and All-muac CFR from the same study. This analysis could only be performed with the reports that differentiated S-both from the single deficit categories.

### Ethical statement

This is a secondary analysis of published data in the public domain. As such no ethical clearance was required.

## Results

The 21 datasets’ characteristics are shown in Table 1 and the derived mortality data in Table 2 [16, 36, 39–41, 44–58]. A brief description and comments on each study are given in Additional file 2. We have divided the datasets into: 1) those that reported the proportion of children fulfilling both anthropometric criteria (S-both) separately from those children with single deficits (S-muac and S-whz) and excluding oedema: 2) those that reported children with S-both separately, but also included an admixture of oedematous patients: and, 3) those that failed to differentiate those with both deficits from those with a single deficit as well as including oedematous cases in most of these reports.

**Table 1:** Characteristics of studies included in the analysis

Ref	Study	Author & year	Country	Setting^a^	Study design	Prospect Retrosp	Date of data	MUAC stand	WHZ stand	Age range	Oed	Oed cases	“Both” in study	“Both” in survey	Time obs	Missing data	Default or lost	Original analysis
#	#						year	mm	Z or %	month		%	%	%	d.m	%	%	
Studies giving numbers of children fulfilling both WHZ and MUAC criteria																		
41	1	Aguayo 2015	India	IPF	Case notes	Retrosp	2009–11	< 115	<− 3 WHO	6–59	excl		63.9	21.3	14 d	ng	22.9	Log reg
42	2	Grellety 2012	Niger	Com	Com trial	Prospect	2010	< 115	<−3 WHO	**6–23**	excl		35.1	24.9	5 m	5.3	0.0	Cox PH reg
21	3	Grellety 2015	South Sudan	OTP	Case notes	Retrosp	2008	< 115	<−3 WHO	6–59	excl		31.7	20.3	51 d	8.6	15.0	Binom reg
43	4	Isanaka 2015	Niger	OTP	RCT	Prospect	2012–3	< 115	<−3 WHO	6–59	excl		39.2	24.9	29.5 d	0.0	1.0	Log-binom
44	5	Lowlaavar 2016	Uganda	IPF	Cohort	Prospect	2012–3	< 115	<−3 WHO	6–60	excl		22.5	11.6	3 d	ng	9.7	Log reg
45	6	LaCourse 2014	Malawi	IPF	Case notes	Prospect	2011–2	**< 110**	< 70% NCHS	6–60	excl		14.2	8.8	ng	0.5	0.0	Chisq OR
35	7	Olofin 2016	DRC,Senegal,Nepal	Com	Com cohort	Prospect	1983–92	< 115	<−3 WHO	6–59	excl		21.9	20,12,7	2–6 m	ng	ng	Cox PH reg
Studies including oedema																		
46	8	Berkley 2005	Kenya	IPF	Case notes	Prospect	1999–02	< 115	<−3 NCHS	**12–59**	incl	34.2	42.9	8.8^**b**^	ng	3.6	ng	ROC curve
47	9	Chiabi 2017	Cameroun	IPF	Case notes	Retrosp	2006–14	< 115	<− 3 WHO	6–59	incl	ng	58.5	17.0	ng	30.7	10.1	ROC curve
48	10	Sachdeva 2016	India	IPF	Case notes	Prospect	2012–3	< 115	<−3 WHO	6–60	incl	3.8	32.2	21.3	3.7 d	ng	15.0	ROC curve
Studies where children fulfilling both criteria are incorporated into both the WHZ and MUAC categories																		
49	11	Burza 2016	India	Com	Follow up	Prospect	2009–11	< 115	<−3 WHO	6–59	ng	ng	ng	21.3	18 m	26.7	ng	Log reg
50	12	Mogeni 2011	Kenya	IPF	Case notes	Prospect	2007–9	< 115	<−3 WHO	6–60	incl	19.2	ng	8.8^**b**^	ng	5.4	ng	Chisq
51	13	Sylla 2015	Senegal	IPF	Case notes	Retrosp	2012	< 115	<− 3 WHO	**0–60**	ng	ng	ng	6.6	ng	ng	ng	Log reg
52	14	Vella 1990	Uganda	Com	Com cohort	Prospect	1988	< 115	<−3 NCHS	**0–60**	incl	ng	ng	11.6	ng	13.0	10.0	Log reg
53	15	Dramaix 1993	DRC	IPF	Case notes	Prospect	1986–8	< 115	< 70% NCHS	0–60	incl	28.9	ng	11.5	60 d	15.4	ng	Log reg
54	16	Girum 2017	Ethiopia	IPF	Case notes	Retrosp	2013–5	< 115	**< 70% ng**	ng	incl	66.6	ng	15.5	13 d	ng	11.6	Cox PH reg
55	17	Savadogo 2007	BFA	IPF	Case notes	Retrosp	1999–03	**Tertile**	**<−3 NCHS**	0- < 36	excl	ng	ng	18.3	19 d	ng	18.3	Cox PH reg
36	18	Garenne1987	Senegal	Com	Com cohort	Prospect	1983–4	< 115	<−3 NCHS	**1–60**	incl	ng	ng	6.6	3 m	ng	ng	Life table
37	19	Garenne 2009	Senegal	Com	Com cohort	Prospect	1983–4	**< 110**	**CDC2000**	**1–60**	incl	ng	ng	6.6	3 m	ng	ng	Life table
37	20	Garenne 2009	DRC	Com	Com cohort	Prospect	1989–92	**< 110**	**CDC2000**	**1–60**	incl	ng	ng	11.5	6 m	ng	ng	Life table
38	21	VD Broeck 1993	DRC	Com	Com cohort	Prospect	1989–92	**<−4 SD**	<−3 NCHS	**0–60**	incl	ng	ng	11.5	3 m	ng	ng	Life table

^a^ For brief description of each study and comments please see Additional File 2

^b^ These studies were on the Kenya coast and the nutritional survey was from North Kenya

*DRC* Democratic Republic of Congo (COD), *BFA* Burkina Faso, *IPF* In-patient Facility, *Com* community study, *OTP* Out-patient treatment program, *RCT* randomized controlled trial, *Retrosp* retrospectiv*e*, *Prospect* prospective, *stand* standards used to define SAM, *WHO* World Health Organisation 2006 WHZ standards, *NCHS* National Center for Health Statistics 1996 standards, *CDC2000* Centre for Disease Control 2000 standards, *Oed* oedematous cases (kwashiorkor and marasmic-kwashiorkor), *excl* excluded from analysis, *incl* included in analysis, *ng* not given in report, *“Both” in study* the proportion of all the SAM cases which had both WHZ < − 3Z and MUAC < 115 mm in the study’s subjects, *“Both” in survey* the proportion of all SAM cases that had both WHZ < − 3Z and MUAC < 115 mm derived from a nutritional survey of a random sample of the community (see [21]), *obs* average time the subjects were observed to determine the outcome, *d.m* days or months, *log* logistic, *reg* regression, *Binom* binomial, *PH* Proportional Hazards, *Chisq* Chi-squared Test, *OR* odds ratio. Non-standard definitions of SAM and age ranges not within the 6-60 month age range are shown in bold script.

**Table 2:** The numbers, mortality and significance of differences by criterion for studies analysed

Ref	Study	Author & year	Kwash		Total SAM		S-whz		S-muac		S-both		*S-whz*	*S-muac*	*S-both*	WHZ v MUAC			
			Total	dead	Total	dead	Total	dead	Total	dead	Total	dead	*CFR*	*CFR*	*CFR*	χ^2^	χ^2^	Fisher	Cramer
#	**#**		#	#	#	#	#	#	#	#	#	#	*%*	*%*	*%*	value	p (2-tail)	p (1-tail)	V
Studies giving numbers of children fulfilling both WHZ and MUAC criteria																			
41	1	Aguayo 2015	231	11	6076	32	1333	3	859	2	3884	27	*0.23*	*0.23*	*0.70*			0.65	0.00
42	2	Grellety 2012	2	0	530	13	177	3	167	2	186	8	***1.69***	*1.20*	*4.30*			0.53	0.02
21	3	Grellety 2015	excl	excl	2205	40	1486	13	21	1	698	26	*0.87*	***4.76***	*3.72*			0.22	0.12
43	4	Isanaka 2015	excl	excl	2399	13	530	3	929	4	940	6	***0.57***	*0.43*	*0.64*			0.48	0.03
44	5	Lowlaavar 2016	excl	excl	262	21	171	11	32	0	59	10	***6.43***	*0.00*	*16.95*			0.14	0.10
45	6	LaCourse 2014	97	8	210	15	29	2	68	4	16	1	***6.90***	*5.88*	*6.25*			0.58	0.02
35	7	Olofin 2016	ng	ng	767	145	116	18	483	64	168	63	***15.50***	*13.25*	*37.50*	0.41	0.52	0.31	0.03
Studies including oedema																			
46	8	Berkley 2005	390	ng	1140	193	267	27	384	42	489	124	*10.11*	***10.94***	*25.36*	0.11	0.74	0.42	0.01
47	9	Chiabi 2017	ng	ng	106	22	23	1	21	3	62	18	*4.35*	***14.29***	*29.03*			0.27	0.17
48	10	Sachdeva 2016	ng	ng	447	59	228	18	75	15	144	26	*7.89*	***20.00***	*18.06*	8.52	**0.004**	**0.004**	0.17
Studies where children fulfilling both criteria are incorporated into both the WHZ and MUAC categories																			
							All-whz		All-muac				*All-whz*	*All-muac*		All-whz v All-muac			
49	11	Burza 2016	ng	ng	650	36	295	26	650	36			***8.81***	*5.54*		3.55	0.06	**0.042**	0.06
50	12	Mogeni 2011	392	ng	1768	217	966	101	802	116			*10.46*	***14.46***		6.54	**0.011**		0.06
51	13	Sylla 2015	ng	ng	272	42	90	27	117	15			***30.00***	*12.82*		9.28	**0.002**	**0.002**	0.21
52	14	Vella 1990	ng	ng	118	22	34	3	96	18			*8.82*	***18.75***				0.14	0.12
53	15	Dramaix 1993	320	102	442	122	267	63	175	57			*23.60*	***32.57***		4.31	**0.038**	**0.025**	0.10
54	16	Girum 2017	363	25	545	51	130	26	277	41			***20.00***	*14.80*		1.74	0.19	0.12	0.07
55	17	Savadogo 2007	excl	excl	1322	212	930	168	387	95			*18.03*	***24.50***		7.19	**0.007**		0.01
36	18	Garenne1987	ng	ng	622	63	92	14	569	54			***15.22***	*9.49*		2.81	0.09	0.06	0.07
37	19	Garenne 2009	ng	ng	281	44	183	23	98	21			*12.57*	***21.43***		3.79	0.051	**0.040**	0.12
37	20	Garenne 2009	ng	ng	388	25	150	9	238	16			*6.00*	***6.72***		0.08	0.78	0.48	0.01
38	21	Broeck 1993	56	8	652	13	113	3	539	6			***2.65***	*1.11*				0.14	0.05

*S-whz* WHZ below cut off point with MUAC above cut-off point as defined in the paper, *S-muac* MUAC below cut off point with WHZ above cut-off point as defined in the paper, *S-both* MUAC and WHZ both below the cut-off point as defined in the paper, *All-whz* WHZ below the cut-off point, with MUAC either above or below the cut-off point as defined in the paper, *All-muac* MUAC below the cut-off point, WHZ either above or below the cut-off as defined in the paper, *excl* excluded from analysis, *ng* not given in report, *CFR* Case Fatality Rate in percentage, *χ*^*2*^ Pearson’s chi squared test (two-way significance), *Fisher* Fisher’s exact test (one-tailed test of significance), *Cramer* Cramer’s V test of association between the groups. The higher CFR and significant differences are shown in bold script

As the current proposal is to cease using WHZ and implement MUAC-only programs, in deciding whether such a policy is ethical, the focus has to be on those children who would then be excluded; that is, children diagnosed by WHZ but have neither the MUAC nor the oedema criteria for SAM. The only studies which make this differentiation possible are those giving data for S-both. This is because the CFR for S-whz cannot be ascertained when the WHZ category also includes S-both children. These are shown in the first two categories (datasets 1 to 10). In order to examine the relative mortality statistically it is necessary to have sufficient deaths in each group. Reports 1 to 7, individually, have insufficient deaths to allow for meaningful differentiation of WHZ from MUAC mortality risk; none were statistically different using Fisher’s exact test.

### The effect of oedema

From our empirical data [33] and other studies [59, 60] it is clear that children with oedema have a higher mortality than those that are oedema free, no matter their anthropometric status. However, the augmentation of oedema related mortality appears to be different in children who have wasting by MUAC or by WHZ.

**Fig. 2:**
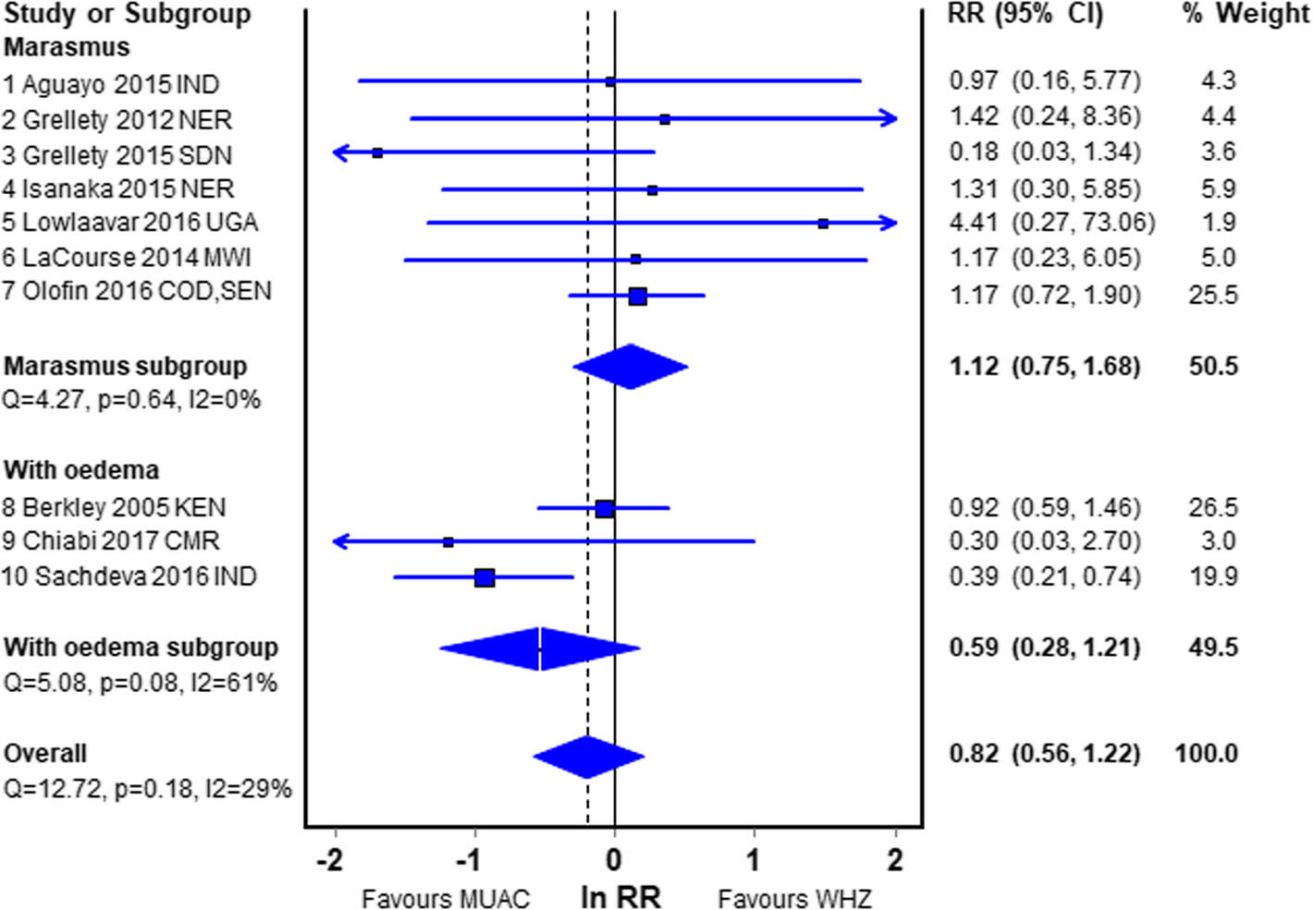
Forest plot of the RR in children diagnosed by WHZ-only relative to MUAC-only with and without oedema. Legend: *IND* India; NER Niger; *SDN* South Sudan; *UGA* Uganda; *MWI* Malawi; *COD* Democratic Republic of the Congo; *SEN* Senegal; *KEN* Kenya; *CMR* Cameroun; Ln *RR* natural log of relative risk; *CI* confidence intervals. In each of the forest plots “favours WHZ” indicates that the Relative Risk for death is higher in children with WHZ < − 3Z than with a MUAC of < 115 mm; “favours MUAC” indicates that the Relative Risk for death of children with a MUAC < 115 mm is higher than those with WHZ < − 3Z

Figure 2 shows a meta-analysis of papers 1 to 10 comparing those with and without admixture of oedematous cases. Although none of the pooled differences are significant, the first 7 studies that have excluded oedematous cases show that the RR of death is greater in S-whz than S-muac (RR, 1.12; CI, 0.75–1.68). For the 3 studies that included oedema the RR indicates that S-muac has a higher mortality risk than S-whz  (RR, 0.59; CI, 0.28–1.21).

### Effect of the reference criteria

**Fig. 3:**
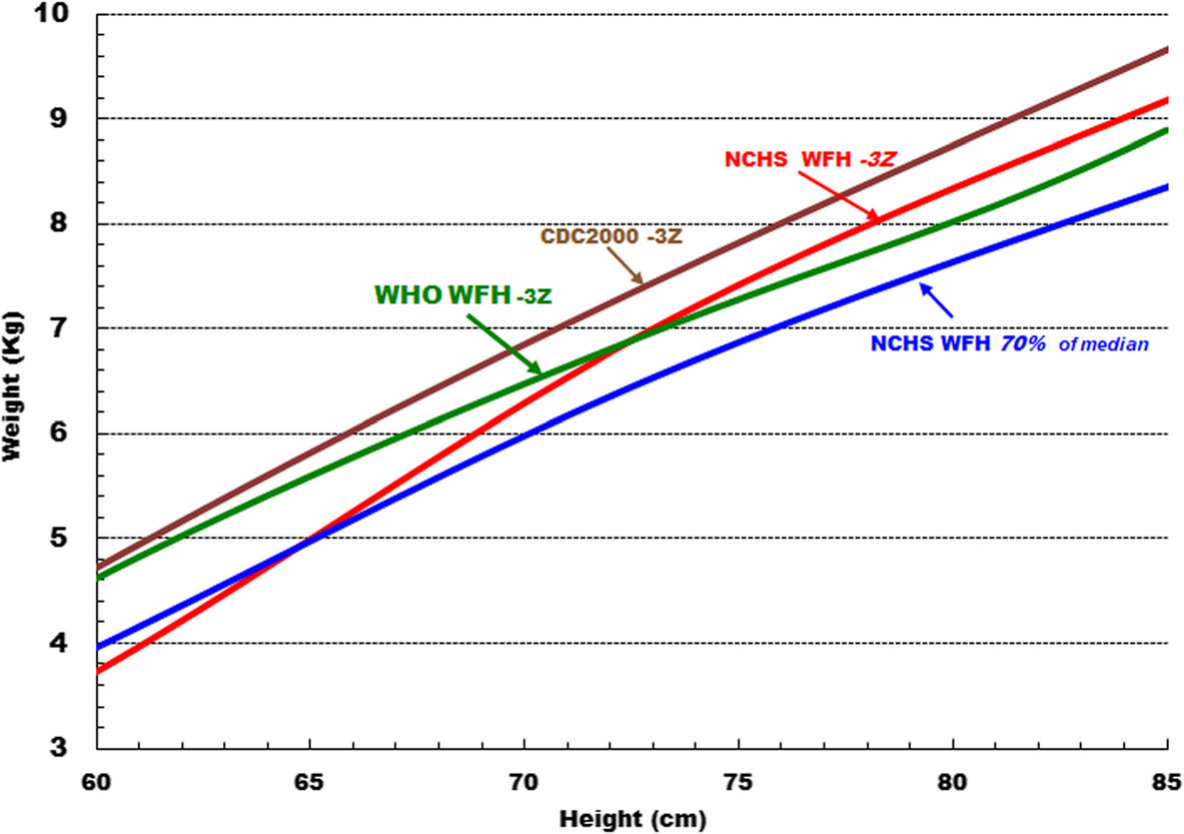
The cut-off weights for heights that define SAM by the different references in use in the studies reviewed. Legend: *WHO* World Health Organisation, 2006 standards; *NCHS* National Center for Health Statistics (USA) 1977; *CDC2000* Center for Disease control and Prevention, Atlanta, USA, 2000 reference

WHO recommends that only the WHO_2006_ standards be used as the reference for WHZ and < 115 mm for MUAC diagnosis of SAM in children 6 to 59 months [61, 62]. Obsolete references were naturally used for studies published before 2006 and these have not, to our knowledge, been re-published using WHO criteria. References that are more stringent than the WHO_2006_ standards, such as the NCHS reference, should have a higher case fatality rate (CFR) and a lower case load than would be the case if the WHO standards were used. This is because the children then diagnosed as SAM will have a lower mean WHZ and thus be more severely malnourished with a higher risk of death than those included with a less stringent reference. Fig. 3 shows the cut-off weights for given heights of the references used in the various studies. Where a more lenient reference, such as the CDC_2000_ curves, is used to define SAM the additional children included in the SAM cohort will have an ameliorating effect on the CFR as they are at a lower mean risk of death, but the additional children will increase the case load. This effect can be quite profound because the absolute number of children included in the SAM category increases exponentially as the diagnostic criteria are relaxed. This is because the shape of the distribution curve of anthropometric parameters in the community is approximately Gaussian [63, 64]. Similarly, where a MUAC of < 110 mm has been used we expect a higher CFR and a lower case-load, than where < 115 mm has been used to define SAM. Thus, whether either the MUAC or WHZ criterion is more or less stringent the relative CFRs and case-loads will change reciprocally. The interpretation of the mortality rates must be judged by the references used. As shown in Fig. 3, the different reference slopes are non-linear. It is not possible to convert data obtained from one set of references to another or to properly amalgamate the data from studies that used obsolete standards.

**Fig. 4:**
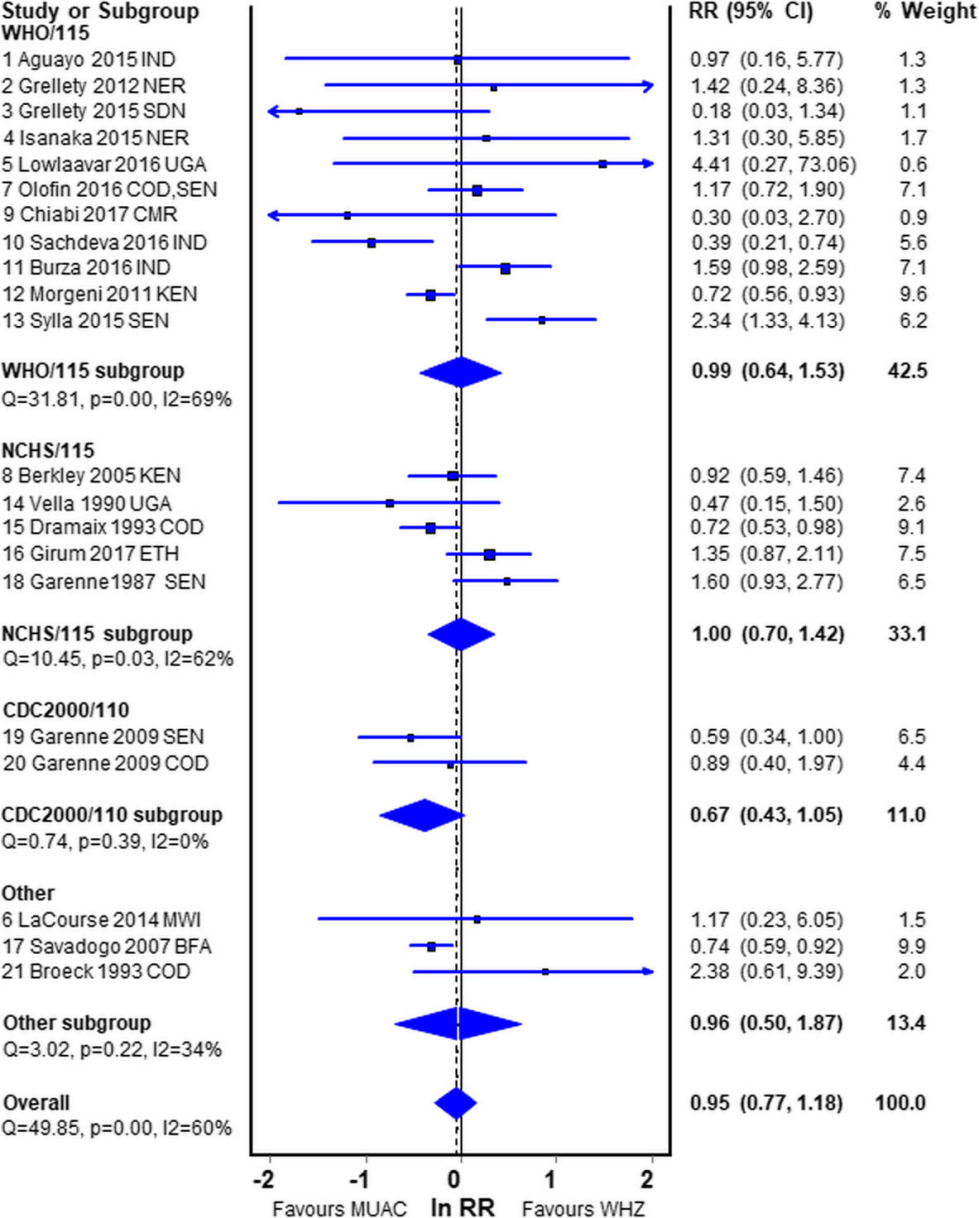
Forest plot of the RR in children diagnosed by WHZ relative to MUAC grouped by the standards used. Legend: *WHO/115* WHO criteria and MUAC< 115 mm; NCHS/115 NCHS criteria and MUAC< 115 mm; *CDC2000/110* CDC2000 criteria and MUAC< 110 mm; *IND* India; *NER* Niger; *SDN* South Sudan; *UGA* Uganda; *SEN* Senegal; *CMR* Cameroun; *KEN* Kenya; *COD* Democratic Republic of the Congo; *ETH* Ethiopia; *MWI* Malawi; *BFA* Burkina Faso; RR relative risk; CI confidence intervals

Figure 4 shows the meta-analysis of all the datasets grouped by the standards that were used. None of the pooled differences showed a significant difference between the risk of death of children fulfilling the MUAC or the WHZ criteria. The studies that used the WHO recommended criteria have an equivalent mortality risk for WHZ and MUAC.

Unexpectedly, those studies that used the NCHS reference had the same RR as those that used the WHO standards with no difference between MUAC and WHZ. Theoretically, WHZ should have had a higher mortality because of the shape of the cut-off for NCHS -3Z; but, as the WHO and NCHS reference lines cross each other at about 73 cm height the net effect will depend upon the height (age) distribution of the children in the studies. The lack of difference between WHO and NCHS could also be due to confounding by inclusion of oedematous children in each of the NCHS studies as well as some of the WHO studies. Nevertheless, there is no net difference and for this reason we have included studies that used the NCHS reference in our main meta-analysis.

The two datasets (a single report) that used the CDC2000 criteria [40] and reduced the cut-off for MUAC to < 110 mm (CDC2000/110) strongly favoured a higher risk of death for those admitted by MUAC; this was expected and almost reached significance. Report 18 and 19, from Senegal, used exactly the same original data, therefore these reports can be directly compared. When NCHS and MUAC< 115 mm (NCHS/115) was used All-whz has a much higher mortality than All-muac (RR, 1.60; CI, 0.93–2.77); whereas, when CDC2000/110 was used All-muac had a much higher risk than All-whz which just reaches significance (RR, 0.59; CI, 0.34–1.00). The same data were also used in reports 20 and 21 from DRC; again there is a substantial difference between the RRs when using the two different anthropometric references. Savadogo et al. [58], in the “other” group, used an obsolete MUAC-for-age formula to calculate All-muac Z-scores [65] and then divided the results into tertiles for analysis; minus 4Z using this standard increases from 103 mm to 111 mm between 6 and 59 months of age. The mean Z score of Savadogo’s children on admission was − 4.59Z which for a 12 to 24 month old child equates to about 100 mm. It is clear that the MUAC standard for these children was very low which would account for the RR of death being significantly greater for S-muac than S-whz (RR, 0.74; CI, 0.59–0.92).

In each of the other meta-analyses we have omitted these reports using CDC2000 reference and MUAC< 110 mm (# 19 & 20), the duplicated data from those reports (# 18 & 21) as well as Savadogo’s report (# 17). The data from datasets 18 to 21 are incorporated into report #7.

### Effect of mode of treatment

**Fig. 5:**
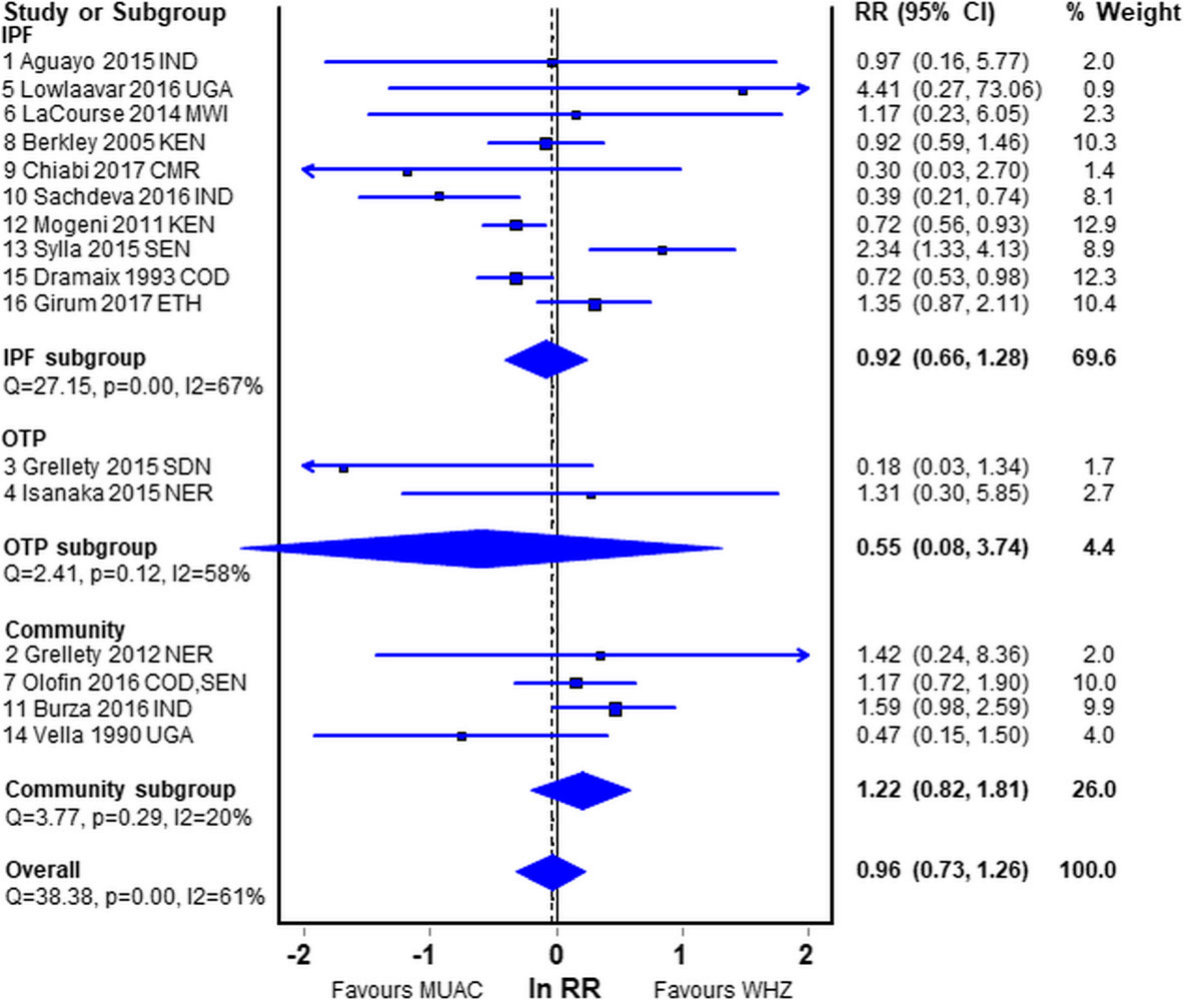
Relative Risk of mortality in children diagnosed by WHZ relative to MUAC by mode of treatment. Legend: *IPF* In-patient Facility (Hospital. Therapeutic Feeding Center); *OTP* Out-patient treatment program (Home treatment); *Com* community study; *IND* India; *NER* Niger; *SDN* South Sudan; *UGA* Uganda; *SEN* Senegal; *CMR* Cameroun; *KEN* Kenya; *COD* Democratic Republic of the Congo; *ETH* Ethiopia; *MWI* Malawi; *BFA* Burkina Faso; RR relative risk; CI confidence intervals

In-patient cohort studies are often criticised on the basis that they do not represent the children in the community and are subject to ascertainment bias [22, 30]. In Fig. 5 we sub-divide the reports by mode of treatment to determine whether this has an effect on comparison of WHZ and MUAC deaths. The in-patients had about the same mortality risk with MUAC and WHZ (RR, 0.92; CI, 0.66–1.28).

The two OTP studies are dominated by a retrospective study from Sudan (study 3) where there were very few S-muac admissions (WHZ 1486 with 13 deaths; MUAC 21 with 1 death); there was no follow-up of defaulters to distinguish death from simple non-attendance. The other report (study 4) from a secondary analysis of a prospective RCT is much more reliable and shows S-whz to have a greater, but non-significant, RR of death (RR, 1.31; CI, 0.30–5.8).

The community studies also showed a non-significant higher risk with WHZ than MUAC (RR, 1.22; CI, 0.82–1.81).

### Overall analysis

**Fig. 6:**
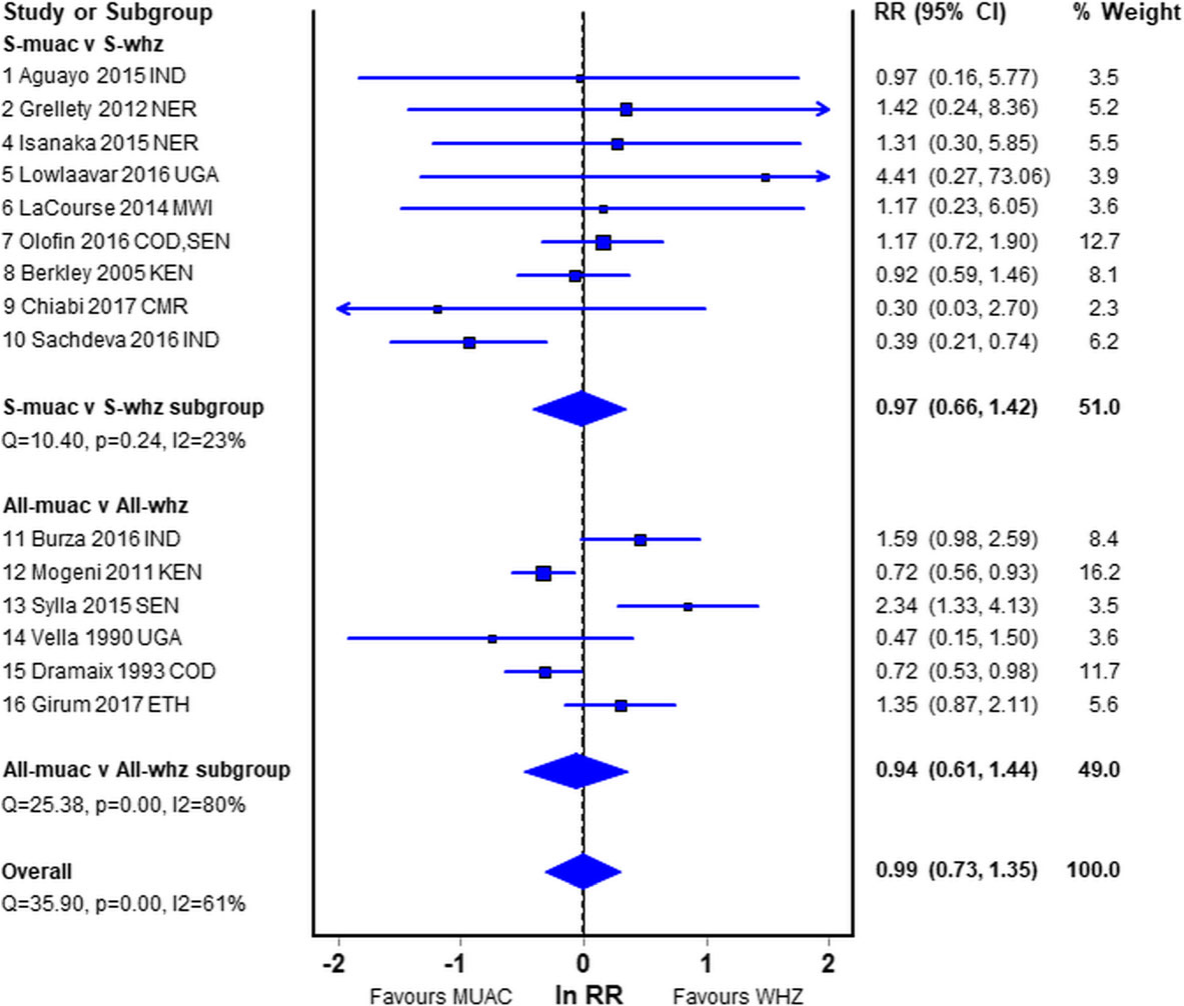
Relative Risk of mortality in children diagnosed by WHZ relative to MUAC omitting the duplicate data. Legend: *S-whz* WHZ below cut off point with MUAC above cut-off point as defined in the paper; *S-muac* MUAC below cut off point with WHZ above cut-off point as defined in the paper; *All-whz* WHZ below the cut-off point, with MUAC either above or below the cut-off point as defined in the paper; *All-muac* MUAC below the cut-off point, WHZ either above or below the cut-off as defined in the paper; *IND* India; *NER* Niger; *SDN* South Sudan; *UGA* Uganda; *SEN* Senegal; *CMR* Cameroun; *KEN* Kenya; *COD* Democratic Republic of the Congo; *ETH* Ethiopia; *MWI* Malawi; *BFA* Burkina Faso; RR relative risk; CI confidence intervals

Figure 6 shows the overall analysis, omitting the duplicated studies found in the previous analyses. Here they are sub-grouped by whether S-both was incorporated or excluded from the analysis. There was no difference in the risk of death between those with a low MUAC and those with a low WHZ (RR, 0.99; CI, 0.73–1.35). The heterogeneity of the S-whz v S-muac group was low (I^2^ = 23%) but high in the All-whz v All-muac group (I^2^ = 80%). The statistics, sensitivity analysis, Doi and funnel plots are given in Additional file 3: Figure S1.

### Grouping of deficits

As there was no difference in the meta-analysis of children with single and double deficits (Fig. 6), and we had found in our empirical data that inclusion of both deficits into the S-whz and S-muac groups gave erroneous results due to mathematical coupling, we examined the difference this change in analytical procedures would cause using the studies where we had information on S-both.

The differences between CFRs of S-whz v S-muac and All-whz v All-muac in reports 1 to 10 are shown in Table 3. In two of the papers the All-whz/muac gave a larger difference in CFR than the S-whz/muac comparison; in 6 of the papers S-whz/muac CFR comparison was larger than the All-whz/muac comparison. In the other 2 papers by Lowlaavar et al. [47] and Berkley et al. [49] the direction of the difference was actually reversed (in opposite directions), so by inclusion of children with both deficits into each of the single groups contrary conclusions would have been reached. These latter studies are examples of Simpson’s paradox, an extreme form of confounding [33, 66], but inclusion of S-both into the whz v muac comparison led to erroneous results in every study.

**Table 3:** The effect of mathematical coupling on the interpretation of CFR and possibility of Simpson’s paradox

Study	Author & year	Single deficits alone					Single and both deficits					Difference WHZ-MUAC		
		*S-whz*	*S-muac*	S-whz v S-muac			All-whz	All-muac	All-whz v All-muac			S-whz-S-muac	All-whz-All-muac	Effect on CFR
		*CFR*	*CFR*	χ^2^	Fisher	Cramer	CFR	CFR	χ^2^	Fisher	Cramer			
		*%*	*%*	*p*	*p*		%	%	p	p				
1	Aguayo 2015	0.23	0.23	*nc*	(0.96)	0.00	0.58	0.61	0.81	nc	0.00	−0.01	−0.04	>All
2	Grellety 2012	1.69	1.20	*nc*	0.73	0.02	3.03	2.83	0.88	0.880	0.01	0.50	0.20	>S
3	Grellety 2015	0.87	4.76	*nc*	(0.19)	0.05	1.79	3.76	0.002	nc	0.06	−3.89	−1.97	>S
4	Isanaka 2015	0.57	0.43	*nc*	(0.68)	0.01	0.61	0.54	0.77	nc	0.01	0.14	0.08	>S
5	Lowlaavar 2016	6.43	0.00	*nc*	0.14	0.10	9.13	10.99	0.61	0.607	0.03	6.43	−1.86	Reverse
6	LaCourse 2014	6.90	5.88	*nc*	0.83	0.02	6.67	5.95	nc	0.860	0.01	1.01	0.71	>S
7	Olofin 2016	15.52	13.25	0.52	0.52	0.03	28.52	19.51	0.002	nc	0.10	2.27	9.01	>All
8	Berkley 2005	10.11	10.94	0.74	0.74	0.01	19.97	19.01	0.63	nc	0.01	−0.83	0.96	Reverse
9	Chiabi 2017	4.35	14.29	*nc*	0.31	0.17	22.35	25.30	0.65	0.659	0.03	−9.94	−2.95	>S
10	Sachdeva 2016	7.89	20.00	**0.004**	**0.005**	0.17	11.83	18.72	**0.021**	**0.023**	0.09	−12.11	−6.89	>S

*S-whz* WHZ below cut off point with MUAC above cut-off point as defined in the paper, *S-muac* MUAC below cut off point with WHZ above cut-off point as defined in the paper, *All-whz* WHZ below the cut-off point, with MUAC either above or below the cut-off point as defined in the paper, *All-muac* MUAC below the cut-off point, WHZ either above or below the cut-off as defined in the paper, *S-whz–S-muac* (single deficit) WHZ minus MUAC CFR, *All-whz – All-muac* (combined deficits) WHZ minus MUAC CFR, *CFR* Case Fatality Rate in percentage; *χ*^*2*^ significance of Chi-squared analysis of MUAC against WHZ CFRs; *Fisher* Fisher’s exact test, two-sided mid *P*-value (values in parentheses are approximate as one cell number too large); *Cramer* Cramer’s V of association between variables; *“>All”* the difference in CFRs between WHZ and MUAC is greater with All-whz/muac; *“>S”* the difference is greater with S-whz/muac; *“Reverse”* the direction of the change is reversed (Simpson’s paradox), negative numbers WHZ < MUAC, positive numbers WHZ > MUAC. Significant differences are shown in bold script

## Discussion

Most of the original analyses in the papers examined considered the whole population of children, either in the community or admitted to the IPF (hospital). They tested the diagnostic ability of MUAC and WHZ to predict the death of the children compared to those that did not fulfil any of the criteria for SAM and then compared statistics such as the areas under a ROC curve. They frequently proposed changes in the cut-off points to maximise the sensitivity and specificity (giving sensitivity and specificity equal importance) of the diagnosis to predict death. Comparison of SAM with non-SAM children was not the objective of our analysis. The WHO has specified the standards and cut-off points that are used to define SAM; we are not examining how appropriate these cut-off points are to define SAM-related deaths or to see how a change is the MUAC cut-off would capture more children with WHZ < −3Z. Our objective was to compare the risk of death of SAM children identified by either the WHO specified MUAC or WHZ criteria directly. Thus, unlike the reported studies, the children who did not have SAM were not considered relevant in our analysis. This is the first review of SAM-related mortality data to be examined in this way.

The most reliable of the meta-analyses is probably the first subgroup shown in Fig. 2. These 7 reports are the only reports where children with S-whz and S-muac were separated in the analyses to eliminate the effect of mathematical coupling [37, 38], oedematous children were excluded, the age range of the children was from 6 to 60 months and the WHO recommended criteria for diagnosis of SAM were used. However, 6 of the 7 studies were under-powered. The pooled result showed no significant difference between the risk of death for those admitted that fulfilled the WHZ criterion and those that fulfilled the MUAC criterion. Nevertheless, the combined data showed that WHZ had a slightly higher mortality risk (RR, 1.12; CI, 0.75–1.68) than children fulfilling the MUAC criterion. The group of studies from the community in Fig. 5, although old and subject to mathematical coupling, should not have an ascertainment bias; they also showed a higher risk with a low WHZ (RR, 1.22; CI, 0.82–1.81). These two groups are probably more reliable than the other studies and are both in agreement with our empirical data showing a higher risk of death for children with WHZ < −3Z than MUAC < 115 mm. These latter community studies are the class of study that is most frequently quoted in favour of a MUAC only program.

When the other studies that are potentially subject to more severe bias are included there was no difference in the relative mortality risks between children admitted by MUAC and those admitted by WHZ (RR 0.99). It is clear that 6–60 month old children with WHZ < −3Z or MUAC < 115 mm both have a substantial risk of death and that children with WHZ < −3Z are at an equivalent or higher risk than those with a low MUAC. There is no justification in labelling children with a WHZ < −3Z and a MUAC > 115 mm as healthy and denying then treatment [22, 23, 25–30] in preference to children with a MUAC < 115 mm, although it is acknowledged pragmatically that MUAC is a much easier and more convenient measure at the present time.

### Potential sources of Bias

The data from the studies included in this review are all subject to bias, some sufficiently severs to render the studies of little value in setting policy. The major problems with some of the studies are outlined in Additional file 2.

### Confounding

#### Oedema

Oedema is clearly a major confounder in some studies. The paper by Girum et al. [57] had 67% of oedematous children in their analysis, and in the paper by Berkley et al. [49], which is very widely quoted, 38% of the children fulfilling the S-muac criterion and 14% of those with S-whz had oedema. It is unclear which way oedema affects the relative mortality of S-muac and S-whz children. In paper I [33] oedematous children had a much higher mortality when associated with WHZ < −3Z, whereas in our meta-analysis oedematous children with a low MUAC had the higher risk of death. We do not have an explanation for this discrepancy.

In the report by LaCourse [48], infants with a low MUAC and oedema had a 21% CFR, compared to 6% in those with only a low MUAC. This large difference may indicate a different prognostic impact of oedema in infants, possibly because sodium homeostasis appears to be different in malnourished children less than 12 months of age from older children [67]. However, oedematous malnutrition normally has a higher incidence in older children. The degree of oedema may also affect the increase in mortality risk [60]. It is thus difficult to predict the relative magnitude of the increase in mortality risks due to oedema in different groups of patients.

The community based studies, which are quoted extensively in support of MUAC-only programs [40, 68–78] did not exclude oedematous cases. As oedematous malnutrition usually has a short history before death (a few days), it will not have been recorded during the antecedent anthropometric measurements and oedema is not usually observed by parents unless it is gross and accompanied by other signs of kwashiorkor. This problem does not arise with the patient based studies.

#### Other co-morbidity bias

Most of the studies do not report signs and symptoms, investigations, infections, complications or other characteristics of their children and how they differ between groups. They rarely report the putative causes of death. Where such data are reported there are always significant differences in the characteristics of the children admitted with S-muac and S-whz apart from age and oedema status. For example, Berkley et al. [49] report significant differences between their groups in degree of stunting, skin/hair signs of kwashiorkor, and gender. Sachdeva et al. [51] report differences in age, fast breathing and duration of illness. But they do not report statistical comparisons between the anthropometric groups, only between those that survived and died.

When the putative causes of death are recorded, it appears that many of the deaths were due to conditions that are not normally associated with SAM and would not be alleviated by treatment with the standard protocols for the management of SAM. For example, in the reports by Sachdeva and Berkley, 30 and 10% of the SAM children had convulsions respectively; these were of unreported/undetermined aetiologies, but are not normally a feature of malnutrition per se [79]. As the proportion with convulsions varied with diagnostic group there is a potential co-morbidity bias in these studies, and also in the studies that did not report the prevalence of complications in the respective groups.

One of the problems of interpreting the community studies is that death occurs remotely in time to the antecedent anthropometry so it is unknown whether and how the children were malnourished, or not, at the time of death, their relative WHZ and MUAC status, whether their deaths were confounded by oedema and whether the death was from non-nutrition related illness. It is noteworthy in reading the reports that a relatively small proportion of all deaths occurred in malnourished children in the community cohorts. For example, the very careful report by Van den Broeck et al. record 87% of deaths as miscellaneous or unknown [41]. The causes of death vary by age and thus are likely to have different MUAC and WHZ profiles. Non-nutritional mortality requires different strategies to prevent death: for example, immunisation, sanitation, maternal services, HIV prevention, etc. The extent to which nutritional treatment would affect non-nutrition related mortality is unknown, but this and many other studies were conducted in malaria hyper-endemic areas. The potentiating effect of malnutrition on malarial mortality is controversial [1, 80, 81].

#### Diagnostic bias (references used)

The use of obsolete standards also has a profound effect. The reports by Garenne [40], Van den Broeck [41] and Savadogo [58] each used very stringent diagnostic cut-offs for MUAC and Garenne’s report a very lenient WHZ reference. This makes these reports unreliable for setting policy. Of course, as we show in paper III [34] the importance of the CFR has to be judged in relation to the case-load. It is noteworthy that by choosing to use obsolete standards in Garenne et al’s 2009 Senegal report they generated a CRF that is much greater with MUAC than with WHZ. Nevertheless, the attributable risk percentage for severe wasting to cause death was 12.1% for WHZ and only 5.7% for MUAC. Attributable risk is a much more important statistic for setting policy than CFR. By their choice of diagnostic criteria the case load changed in a reciprocal fashion to the CFR. The CFR quoted in Garenne et al’s 2009 paper must not continue to be used to advocate for a MUAC-only policy. Despite this, papers which used obsolete criteria for both WHZ and MUAC, and other biases, are misleadingly quoted as evidence in favour of a MUAC only program [39–41, 48, 49, 55, 56].

#### Ascertainment bias

When focusing upon SAM, the whole community is not relevant. What is relevant is the extent to which the children in the study represent the children *with SAM* in the community. The least biased sample of such children should come from those children with SAM in a large random sample of the community. Although with demographic, social and nutritional change, historical cohorts may not represent SAM in present day circumstances or in other countries, apart from Senegal and the Democratic Republic of Congo, as the diagnosis of SAM by the two criteria differs markedly from place to place [21].

The patients selected in IPFs and OTPs that satisfy both the WHZ and the MUAC criteria are more at risk and are less representative of SAM in the community than those that satisfy a single criterion. We include in Table 2 and Additional file 1: Table S1 a measure of this discordance between the ratios of S-both to total SAM found in the study and in representative samples of SAM in the community [21]; we consider this as an indication of the extent of ascertainment bias. This bias will be much greater if the severest children (those with both criteria) are included in the analysis of patients. We maintain that one reason for splitting the children into those with single deficits is that the S-whz is more likely to be representative of the S-whz children in the community than total SAM in a patient cohort is to total SAM in the community. This is because children with S-both are common in the patient groups and much less common in the community.

The reports from patient cohorts in both IPFs and OTPs have been criticised because they “do not represent the community” and have been dismissed as valid evidence by some [22, 30]. When we eliminate the studies using inappropriate criteria, it is noteworthy that the community derived cohorts have a higher risk of death for WHZ than MUAC diagnosed children (Fig. 5), and not the reverse as those advocating MUAC-only programs speculate [29, 30].

### Verification bias

#### Length of observation

Two of the studies, Lowlaavar [47] and Sachdeva [51], had extremely short observation periods for children in hospital before they died or were discharged. They also have a very high mortality which calls into question the severity of SAM, the admission criteria and the quality of treatment given. It is likely that only children brought directly for clinical care by the parents because of severe complications, and were admitted in extremis*,* form the bases for these two reports. Such children may not be appropriate to guide admission policy for less critically ill children from the community or elsewhere. This is because it is unknown whether children who die with a low MUAC or WHZ have a different time course to death after presentation to the health services. Varied time of observation for each child at risk is a potential source of verification bias.

#### Missing data and defaulting

Missing data is a potential source of bias. Two studies [50, 52] had over one quarter of their data missing. Unfortunately, 9 of the other 18 reports did not give any indication of the extent of missing data.

Defaulting is another source of verification bias. In IPF this is less serious as children that die from SAM usually die early after admission (about 70% of deaths occur in the first week), and most defaults occur later when the caretakers themselves consider that the child has sufficiently recovered, that they are not making any progress or competing priorities at home mount and demand the mother’s attention.

Of more concern is defaulting as outpatients (OTP). There is very rarely any follow up to determine the outcome of children who simply do not return for treatment, so it is not known what proportion of these children “default” because they are actually dead. The CFR for the OTP studies must be seen as a minimum and not an actual death rate. This can lead to very substantial verification bias. OTP study number 4, by Isanaka et al. [46] did not suffer from this defect.

### Mathematical coupling

From our empirical data [33] it is clear that, in judging MUAC and WHZ as independent criteria for diagnosis of SAM, the children with both deficits have a significantly augmented mortality risk. This is confirmed by most of the studies which report the outcome of children with both deficits and have sufficient events (Table 2). Many studies only counted children with a low MUAC and added to that group those that also had a low WHZ (S-both), and their deaths, without separating those with double defects (datasets 11 to 21).

This incorporation of exactly the same data into both groups, and then comparing the groups, results in a phenomenon termed mathematical coupling [37, 38] that causes the analytical results to be in error. In other words some of the children were compared with themselves. The meta-analysis did not show any difference between these two types of selection of children to include in an analysis. We therefore examined the papers where we could assess the extent to which this may have affected the data (Table 3) and found that CFRs were different in each case, some with greater augmentation of WHZ and others of MUAC; two of the studies even reversed the relative magnitude of the WHZ and MUAC mortality rates, a phenomenon termed Simpson’s paradox [66]. The degree and direction of the changes depend upon the relative mortality in the data common to both groups and the relative size of the three groups. These factors were different in each of the studies shown in Table 3.

The report by Lowlaavar [47] is particularly illustrative of the errors that can occur. There were no deaths at all in the children with S-muac and a CFR in the S-whz children of over 6%. When the children with both a low MUAC and WHZ who have a high CFR (17%) are added to the analysis of both groups there is a dramatic reversal so that All-muac now appears to have a higher CFR than All-whz. This analytical error affected most of the studies reported, including those where single defects were also reported, because, in every report the combined data was used in their life tables, logistic (or other) regressions and generated ROC curves. As Tu et al. [66] state: “Incorrect use of statistical models might produce consistent, replicable, yet erroneous results” this seems to be the case with all the original analyses in the literature reviewed.

### Limitations

All the studies reviewed have limitations that are presented in the discussion and in Additional file 2. Community representative samples of children 6–59 months show that only an average of 16.5% of the children meeting the criteria for SAM have both a MUAC and WHZ below the WHO cut-off points; the rest of the SAM children have either one or the other criterion making them eligible for treatment [21]. The studies reviewed all have a higher proportion of children satisfying both criteria than are present in the community. In Table 1 we show the overlap (S-both) of children in the study, and that found in recent representative samples of the community.

The heterogeneity of the standards, the admixture of oedematous cases and the failure to account for confounding, such as TB, HIV, and non-nutrition related conditions, makes simple amalgamation of data from the different study populations problematic. Similarly, there are major co-morbidity, temporal and stochastic biases with the community studies [82]. The community studies were performed at a time when most countries had a much higher prevalence of malnutrition, all-cause mortality, and poor coverage of other public health programs such as measles vaccination, vitamin A capsule distribution, salt iodisation, insecticide impregnated bed-nets and HIV services. It is perhaps for this reason that the community studies also have a higher proportion of children satisfying both criteria than are found with recent surveys in the same areas. It is also probably the reason why the attributable fraction of death due to SAM in these older community studies was lower than expected. It is estimated that SAM and moderate acute malnutrition underlie up to half of all paediatric deaths [80].

The meta-analyses are only as good as the studies that are incorporated into the analysis. We have eliminated the most egregious of the studies, but it must be acknowledged that none of the studies are without potential bias. Therefore, unfortunately, there are no definitive unflawed data upon which to rely in order to unequivocally inform public health policy.

## Conclusions

None of the datasets reviewed, with the possible exception of our empirical data [33] were large enough to have sufficient power to distinguish between the CFRs associated with the WHO criteria for SAM by MUAC and WHZ. They used inappropriate standards, included children outside the age range used by WHO’s SAM treatment protocols [83], included children with oedema or entered individuals with both defects into each of the groups being compared. All the patient studies have an ascertainment bias. None of these studies provide sufficient evidence to support the assertions made by those seeking to drop WHZ as a routine diagnostic criterion for SAM.

The assertions of Briend et al. [22, 26, 31], in particular, that MUAC is consistently “superior” to WHZ as a prognostic indicator, that children with a low WHZ are healthy and do not have an independent mortality risk, and that the two parameters are not additive are all incorrect. Although papers, with completely inadequate data, have been heavily criticised [84], they are still being used to justify a MUAC-only policy [85]; these papers are reviewed here and are found sufficiently problematic that they should not be used to guide policy decisions.

The conclusions drawn from our empirical data [33] are supported by the published reports comparing the prognostic value of WHZ and MUAC. That is that children with a WHZ of <−3Z have about the same or higher risk of death as children with a MUAC < 115 mm. They both are at substantial risk of death, and neither should be omitted from protocols aimed at diagnosis and treatment of all SAM cases.

The relative case loads not only differ between countries, but also determine the absolute number of deaths that occur in each of the diagnostic groups. This is explored in detail in paper III [34]; it has not been sufficiently considered by those advocating for abandonment of WHZ as a diagnostic criterion for SAM.

## Additional files

Additional file 1: **Table S1.** Criteria used for assessing studies quality and risk of bias. (DOCX 17 kb)

Additional file 2: Comments on the individual studies. (DOCX 33 kb)

Additional file 3: **Figure S1.** Statistics, Sensitivity analysis, DOI plot and funnel plot corresponding to Fig. 6. (TIF 2826 kb)

## Abbreviations

CFR: Case fatality rate
IPFs: In-patient treatment facilities
MAM: Moderate acute malnutrition
MUAC: Mid-upper-arm-circumference
OTPs: Out-patient treatment programs
ROC curve: Receiver operating characteristic curve
RR: relative risk
SAM: Severe acute malnutrition
SFCs: Supplementary feeding centres
WHO: the World Health Organisation
WHZ: Weight-for-height Z-score
