# Supplementary material for: Severely malnourished children with a low weight-for-height have similar mortality to those with a low mid-upper-arm-circumference: II. Systematic literature review and meta-analysis
Source: Nutr J. 2018 Sep 15;17:80. doi: 10.1186/s12937-018-0383-5 (PMC6138903; doi:10.1186/s12937-018-0383-5)
Supplement: Supplementary file 1 — Table S1. Criteria used for assessing studies quality and risk of bias. (DOCX 17 kb) [file 12937_2018_383_MOESM1_ESM.docx]

**Additional file S1: Table S1.** Criteria used for assessing studies quality and risk of bias

| **Bias assessment** | **Ascertainment bias** | | **Inclusion/exclusion bias** | | **Confounding** | **Math coupling** | **Age bias** | **Selection bias** | **Observ bias** |  |
| --- | --- | --- | --- | --- | --- | --- | --- | --- | --- | --- |
|  | **Q1** | **Q2** | **Q3** | **Q4** | **Q5** | **Q6** | **Q7** | **Q8** | **Q9** | **Overall quality score** |
| **Criteria** | **Study type** | **Discordance** | **WHZ stan** | **MUAC cut-off** | **Oedema incl** | **Both incl** | **Age gp** | **Missing data** | **Time obs** |  |
| **Coding strategy** | Comm pro = 3 | 1-2 = 1 | WHO = 2 | <115 = 2 | No = 2 | No = 1 | 6-60 = 2 | <5% = 2 | >28 = 2 |  |
|  | Comm retro = 2 | >2 = 0 | NCHS = 1 | <110 = 0 | Yes < 10% = 1 | Yes = 0 | 0-60 = 1 | <15% = 1 | 10-28 = 1 |  |
|  | IPF/OTP pro = 1 | Not given = 0 | CDC = 0 | Other = 0 | Yes > 10% = 0 |  | <23 = 0 | >15% = 0 | <10 = 0 |  |
|  | IPF/OTP retro = 0 |  |  |  | Not given =0 |  |  | Not given = 0 |  |  |
| 1 Aguayo 2015 IND | 0 | 0 | 2 | 2 | 2 | 1 | 2 | 0 | 1 | 0.67 |
| 2 Grellety 2012 NER | 3 | 1 | 2 | 2 | 2 | 1 | 0 | 2 | 2 | 1.00 |
| 3 Grellety 2015 SDN | 0 | 1 | 2 | 2 | 2 | 1 | 2 | 0 | 2 | 0.80 |
| 4 Isanaka 2015 NER | 1 | 1 | 2 | 2 | 2 | 1 | 2 | 2 | 2 | 1.00 |
| 5 Lowlaavar 2016 UGA | 1 | 1 | 2 | 2 | 2 | 1 | 2 | 1 | 0 | 0.80 |
| 6 LaCourse 2014 MWI | 1 | 1 | 1 | 0 | 2 | 1 | 2 | 2 | 0 | 0.67 |
| 7 Olofin 2016 COD,SEN | 3 | 1 | 2 | 2 | 2 | 1 | 2 | 0 | 2 | 1.00 |
| 8 Berkley 2005 KEN | 1 | 0 | 1 | 2 | 0 | 1 | 2 | 2 | 0 | 0.60 |
| 9 Chiabi 2017 CMR | 0 | 0 | 2 | 2 | 0 | 1 | 2 | 0 | 0 | 0.47 |
| 10 Sachdeva 2016 IND | 1 | 1 | 2 | 2 | 1 | 1 | 2 | 0 | 0 | 0.67 |
| 11 Burza 2016 IND | 2 | 0 | 2 | 2 | 0 | 0 | 2 | 0 | 2 | 0.67 |
| 12 Mogeni 2011 KEN | 1 | 0 | 2 | 2 | 0 | 0 | 2 | 0 | 0 | 0.47 |
| 13 Sylla 2015 SEN | 0 | 0 | 2 | 2 | 0 | 0 | 1 | 0 | 0 | 0.33 |
| 14 Vella 1990 UGA | 3 | 0 | 1 | 2 | 0 | 0 | 1 | 0 | 2 | 0.60 |
| 15 Dramaix 1993 COD | 1 | 0 | 1 | 2 | 0 | 0 | 1 | 0 | 2 | 0.47 |
| 16 Girum 2017 ETH | 0 | 0 | 1 | 2 | 1 | 0 | 1 | 0 | 1 | 0.40 |
| 17 Savadogo 2007 BFA | 0 | 0 | 1 | 0 | 2 | 0 | 0 | 0 | 2 | 0.33 |
| 18 Garenne1987 SEN | 3 | 0 | 1 | 2 | 0 | 0 | 1 | 0 | 2 | 0.60 |
| 19 Garenne 2009 SEN | 3 | 0 | 0 | 0 | 0 | 0 | 1 | 0 | 2 | 0.40 |
| 20 Garenne 2009 COD | 3 | 0 | 0 | 0 | 0 | 0 | 1 | 0 | 2 | 0.40 |
| 21 Broeck 1993 COD | 3 | 0 | 1 | 0 | 0 | 0 | 1 | 0 | 2 | 0.47 |

Q1 Study type: Comm = community cohort study, pro = prospective, retro = retrospective, IPF = In-patient facility, OTP = Out-patient program.

Q2 Discordance: this is a ratio of the proportion of the study's children with SAM that satisfy both WHZ and MUAC criteria vs the proportion found in random samples of SAM children in community (i.e. if the study's discordance is 30% and the community's discordance is 20%, then "discordance" would be 30/20 = 1.5).

Q3 WHZ stan: the standards used in the study to define SAM by weight-for-height.

Q4 MUAC cut-off: the absolute MUAC (mm) to define SAM in the study.

Q5 Oedema incl: whether oedematous children were excluded from the study or not and the percent of included children that had oedema.

Q6 Both incl: whether S-muac and S-whz data were obtainable or whether only ALL-muac and ALL-whz were presented. Statistical mathematical coupling error.

Q7 Age group: the age range of children included in the study. Children less than 6 months have a higher mortality rate and children over 23 months a lower mortality rate which both bias the sample towards MUAC having a higher mortality as the WHO criteria for diagnosis of SAM only applies to 6-60 month old children.

Q8 Missing data: This is a minimum total missing data. For community studies, lost to follow up, refusal to participate, non-recording. For SAM patient studies includes sum of missing records/data, default rate and transfer out of the service for medical reasons.

Q9 Time obs: Number of days of observation that the subjects/patients were at risk of death, very short observation times will miss later deaths.

Overall quality: Sum of the "score" for each question divided by the highest score.

No adjustment to score made for possible dehydration
