# Supplementary material for: Severely malnourished children with a low weight-for-height have similar mortality to those with a low mid-upper-arm-circumference: II. Systematic literature review and meta-analysis
Source: Nutr J. 2018 Sep 15;17:80. doi: 10.1186/s12937-018-0383-5 (PMC6138903; doi:10.1186/s12937-018-0383-5)
Supplement: Supplementary file 2 — Comments on the individual studies. (DOCX 33 kb) [file 12937_2018_383_MOESM2_ESM.docx]

## Additional file S2: Comments on the individual studies

**Paper 1, Aguayo et al 2015** [1]. This was a retrospective study from three states of India reporting on the outcome of patients admitted for two weeks to nutrition rehabilitation centres. The CFR was very low. About 64% of the children had both WHZ <-3Z and MUAC <115mm. This is quite unlike nutritional surveys in India showing a major degree of ascertainment bias. Of the 32 deaths there were only 3 with WHZ-only and 2 with MUAC-only the rest had both deficits. This situation makes mathematical coupling extreme. The odds ratio for death of those with oedema was 7.0 (presumably compared to those without oedema). Oedema was excluded from the analysis, but the rest of the cohort by ROC curve comparison. Inclusion of these oedematous children would have led to further major confounding.

**Paper 2, Grellety et al 2012** [2]. This was a prospective community study. The original analysis compared subjects that did and did not register to receive mass supplementation. The data are derived from all the children in 20 randomly selected villages longitudinally measured by independent research teams, lost data was very low and follow up complete. Height was used as a proxy for age and was designed to only select children 6 to 23 months of age. This was a very well conducted study, but again there were insufficient deaths to have the power to differentiate WHZ and MUAC deaths.

**Paper 3, Grellety et al** **2015** [3]. This was a retrospective analysis of children admitted to OTP in South Sudan. MUAC was only measured in children over 65cm (the height of a normal child of 6 months). The population is noted for being very tall and thin. The WHZ group was very large and the MUAC group very small. There were only 21 subjects with one death in the MUAC-only category. However, the MUAC-only mortality rate was greater than the mortality of children with both MUAC <115 and WHZ<-3Z. This is very unusual and calls into question the reliability of the MUAC-CFR which is dependent upon a single death in a small group.

**Paper 4, Isanaka et al 2015** [4]. This is a secondary analysis of SAM children admitted to a randomised control trial of antibiotics (amoxicillin vs. placebo) in out-patient care of SAM. The study was very well conducted with full verification of all deaths. There were few deaths so the comparison of WHZ and MUAC deaths is underpowered.

**Paper 5, Lowlaavar et al 2016** [5]. This was a study of SAM children with proven or suspected infection. Non-infected SAM children were excluded. The objective was to develop a prediction model of in-hospital death among children admitted with proven or suspected infectious diseases of any aetiology; many clinical and biochemical parameters were collected. HIV prevalence was 5.1%. The mean time to death was 2 days and the average length of stay only 3 days. 9.7% of the patients defaulted from the hospital. Children with both defects had a very high mortality; however, there were no deaths in the group with only MUAC <115mm. The data, are subject to Simpson’s paradox.

**Paper 6, LaCourse et al 2014** [6]. This was a study to determine if lay screeners for SAM in hospital could improve the recognition and diagnosis of SAM cases by hospital staff. The screeners took anthropometry from children on presentation. The study was complicated by a change in the standards being used during the study (NCHS to WHO for WHZ and 110mm to 115mm for MUAC). Although oedema was excluded, those with oedema had a very high mortality rate (6/20 children – 30% of those with oedema and MUAC < 115mm died). Inclusion of these oedematous children would have led to major confounding. Other co-morbidities were not reported. Determination of numbers of children and deaths was from a Ven diagram. The mortality for S-both was slightly less than S-whz mortality which is very unusual.

**Paper 7, Olofin et al 2016** [7]. This is a presentation and abstract which amalgamates the original data from 3 community studies in DRC, Senegal and Nepal. The analysis is appropriate, addresses the issue of MUAC and WHZ CFR compared to non-SAM children. The WHO criteria are used for SAM. The authors of this review have had access to the original analytical methods for verification by personal communication. We understand that the full data are now being prepared for publication. The data for Senegal is the same as that used in papers 18 and 19, and the data for DRC is the same as in papers 20 and 21. The duplicate data in the other reports were analysed using obsolete standards and are only included to demonstrate the effect of using different standards. The meta-analysis grouping by standards was performed with these additional studies included in the analysis (where the RR can be found); they were excluded as duplicate data from all other meta-analyses.

**Paper 8, Berkley et al 2005** [8]. This was a well reported review of SAM in a Wellcome Trust supported hospital in Kenya specifically comparing WHZ with MUAC. The ROC curves for the two indices crossed and were almost identical. It is reported that different children died in each group, which is similar to the conclusions of our empirical data, yet interpreted differently to favour using MUAC only. There were major differences in co-morbidity; oedema was confounding in 38% of MUAC-only, 42% of “both” but only 14% of WHZ-only cases. “Seizures” occurred in 7% MUAC-only, 4% of “both”, but 24% of WHZ-only. HIV was not assessed in sufficient numbers to make a comment. It is unclear what the seizures were due to – cerebral malaria, febrile convulsions, hypernatremia are the probable causes, but seizures occurred in 31% of non-malnourished children which would indicate that these were not related to nutritional status for WHZ, but those will a low MUAC may be “protected”. When reported, co-morbidity biases are present in all such studies of malnutrition.

**Paper 9, Chiabi et al 2017** [9]. This small retrospective study has been heavily criticised [10]*.* This is on the basis of massive amounts of missing data found in the authors other publications (data omitted from this publication), a high HIV rate, mathematical coupling to an extraordinary extent and few deaths. The difference in ROC derived from all patients is used to make a strong statement in favour of MUAC-only.

**Paper 10, Sachdeva et al 2016** [11]. This is the only paper advocating for MUAC-only where there were actually more deaths in the children with WHZ-only (18) than with MUAC-only (15) illustrating the importance of case load and presenting attributable risk as well as CFR. In this paper the MUAC-only CFR was higher than those with both deficits (20% v 18%). The mortality rates were very high despite the fact that the length of stay was only 3.7days. This, indicates that either the children were in extremis on admission or that there were problems with the treatment given, possibly with iatrogenic deaths; either of these problems could bias the results. There was a 15% defaulting rate despite the short length of stay. 63% of WHZ, 23% of MUAC and 9% of “both” children had seizures (falciparum malaria not present) indicating major problems with co-morbidity bias. This paper has been quoted in favour of MUAC-only programs [12-14]

**Paper 11, Burza et al 2016** [15]. This study was undertaken when it was found that there was an excessive default rate from an OTP in Bihar, India. They followed up the defaulters and took longitudinal anthropometry of this unusual cohort in the community. All the subjects had been initially admitted to the OTP program on a MUAC-only basis. The cohort was therefore a highly selected, very high risk, community cohort. There was 27% missing data, which makes the study problematic. In this population the CFR for children with a MUAC 110 to 115 was *not* different from non-SAM children, and rose to 5% for 105-110, 10% for 100-105 and >25% of <100mm. There was a very marked effect of season on both relapse and death. This is the only study were a seasonal effect was looked for. It is likely that season is also likely to be a cofounder in other studies, particularly OTP where the patients are at home and exposed to the same environmental and nutritional stresses as the rest of the family during food-insecure times. The degree of sharing of the therapeutic foods, within the family, is also likely to be determined by the food-security status as well as the age of the child. There are well known seasonal effects on the rate of height and weight gain (affecting weight and height at different seasons); there is less of a seasonal effect on MUAC [16]

**Paper 12, Mogeni et al 2011** [17]. This study attempts to compare visible severe wasting with anthropometry for the diagnosis of SAM. The study took place in two hospitals but in only one, the same hospital as paper 8, was WHZ taken, the other appears to be a MUAC-only hospital. The mortality was very high (MUAC 16.8%, WHZ 8.9%), but data for “Both” is not given. 19% of the MUAC children were oedematous and 12% of the WHZ children. The data given for the same facility in paper 8 has mortality rates of about 10% for those with single deficits and 25% for those with both deficits. If the mortality ratio remained the same between the two studies, it appears that a higher proportion of the MUAC children had both deficits than the WHZ children. This would bias the sample and make interpretation of any WHZ v MUAC difference problematic.

**Paper 13, Sylla et al 2015** [18]. This analysis of case notes from a hospital in Senegal aimed to update their hospital records 10yrs after their first audit. In this study there was a remarkably high mortality for WHZ children (30%), but not for MUAC children (8.8%). The age range was from birth with 29% of the patients below the age of 1 month. They found that “dehydration” was strongly associated with mortality. However, this diagnosis was made only on the basis of sunken eyes, skin pinch and lethargy. None of these signs are reliable indicators of dehydration in SAM; this is because in SAM subcutaneous fat loss and dermal atrophy causes the same signs to appear in SAM children who are not dehydrated which is particularly a problem in WHZ children. In fact, they record 21.7% as having these signs (interpreted as dehydration) yet only 15% had any form of diarrhoea so it seems that there was substantial misdiagnosis. If the children with sunken eyes were treated for dehydration, when they were not dehydrated, this would account for the very high mortality in the WHZ children as SAM children are sodium sensitive and readily go into heart failure with excess Na intake (the treatment for truly dehydrated children) Iatrogenic deaths (perhaps up to 15% of WHZ children) could account for the difference observed and cause the data to be biased in favour of a high CFR in WHZ children.

**Paper 14, Vella et al** [19]. There are two reports of this study [20,21] as well as the thesis. The reports appear to give different results in the tables (e.g. in reference 20 it states that in NW Uganda the mortality with WHZ <-2Z (not <-3Z) was 4/32 and in SW Uganda it was 12/142, but MUAC was not taken in NW Uganda. However, in reference 21 only SW Uganda data are given and the reported WHZ mortality is 4/34 for children WHZ <-3Z.). The thesis make the data clear and shows reference 21 to be the most informative.

There is a very steep rise with MUAC mortality <105mm (15/41 =36.6%) but much less from 105-115 (3/55 =5.5%). This pattern of the rise in CFR is unusual. It is also unusual to have 41 subjects with a MUAC <105mm and only 55 in the category 105-115mm. This is because the absolute MUAC follows a Gaussian distribution so either there should have been far fewer children in the <105mm category or more in the 105-115 category. Measurement error, non-random selection of a community cohort, subject selection or data cleaning methods could account for this difference. The WHZ <-3Z mortality was 11.8%. The analysis reported in the paper compared children with a MUAC <105 with WHZ <-2Z. It is not appropriate to compare very severe SAM (by MUAC) with moderate malnutrition (by WHZ) to reach conclusions regarding the relative CFRs using WHO standards and criteria for diagnosis of SAM.

**Paper 15, Dramaix et al 1993** [22]. This is an early study of clinical and biochemical correlates with mortality in SAM children. Their ages ranged from birth. Those with oedema had a high mortality rate; 29% of the whole cohort had oedema; 32% of the oedematous children died. They were followed up to 60 days after discharge. Serum Albumin was the best prognostic indicator followed by sub-scapular skin fold thickness. MUAC mortality increased below 135mm. Compared to Bursa et al (paper 11) this brings into question the appropriateness of one cut-off for absolute MUAC for all populations. In India perhaps 110mm is appropriate and in DRC 135mm is more appropriate. It would be useful to examine these data again without the oedematous cases, and differentiate S-muac for S-both.

**Paper 16, Girum et al 2017** [23]. This is a retrospective analysis of a random sample of case notes from 3 (of 41) hospitals and health centres in Northern Ethiopia, which catered for in-patients. If there were any missing data the case was not used; 4% of case notes could not be found, but the number rejected for missing data is not given. 66.6% of the patients had oedema, which were not separately analysed; half the deaths occurred in oedematous children. Of the subjects 4.4% were less than 6 months. CFR was 9.3%. The admixture of oedematous cases makes the analysis problematic, both in terms of risk of death and the accuracy of the anthropometry in light of the weight of oedema.

**Paper 17, Savadogo et al 2007** [23]. This is a retrospective analysis of children admitted to hospital with SAM. Oedematous cases were excluded. There was a high defaulting rate and 10.9% of the patients were discharged from hospital with a WHZ <-3Z (the admitting criteria) with no data on follow up or the subsequent outcome of these children. The effect of MUAC on mortality was assessed in tertiles of admitted patients. The MUAC z-scores had been assessed using the NCHS 1987 data which are obsolete. The mean Z-score of this patient group was -4.93Z using these standards. This equated so an absolute MUAC of about 100mm. Such a very stringent MUAC cut-off will make the MUAC CFR very high and the data unrealistic to compare with children admitted using the WHO standards. The data are unusable for the purposes of the present review.

**Paper 18, Garenne et al 1987** [25]. This is the original report of the community study in Senegal that has been reanalysed in paper 19 and used in the dataset of report 7. It is a long, very detailed and important report (that has been reprinted with tables now at the end of the document). The tables of interest are 5.2 (p 148), 5.5, 5.7, 5.11, 5.15 and 5.19-22. Oedema is not mentioned. There is no indication of missing data or simple tests of quality of data (for example in how many subsequent visits did a child loose height?). The relative risk with low MUAC increases steadily to 27-32 months of age and is not dominated by infants, which is often assumed. Attributable risk increases to be highest in the 18-59 month old groups – and the RR increases for MUAC, WHZ and HAZ pari-passue. There were deaths from measles, whooping cough and diarrhoea. Tables 5.21 and 5.22 are also of interest as they give the deaths per season. This was worse in the wet season, but appears to be differentially important for the various anthropometric indicators. Perhaps, MUAC or WHZ become dominant, in terms of mortality risk, at different seasons of the year (see comment in paper by Burza) – a possibility that has not been subsequently considered or examined.

**Datasets 19 & 20, Garenne et al 2009** [26]. This paper is a reanalysis of the data originally presented in papers 18 and 21. The objective of the re-analysis was to determine the incidence, duration and CFR of SAM relative to the prevalence. The cut-off values to define SAM in the paper were chosen so that the MUAC cut off (<110mm) was more stringent than the WHO cut-off (<115mm) and the WHZ cut-off (CDC2000) was much more lenient than the WHO standards (fig 3). Otherwise the data used was as reported in the original publications. There is no indication of the numbers of children satisfying both criteria and they were not analysed separately. Although the CFR was higher for MUAC than for WHZ (as expected from the definitions of SAM), the attributable deaths due to WHZ = 12.1% and due MUAC = 5.7% in Senegal. The corresponding figures for DRC were 9.4 and 11.6% both with mortality of 6.2 to 6.6%.

**Paper 21, Van den Broeck et al 1993** [27]. These data are also included in paper 7, where WHO standards are used and oedema is excluded. This is a longitudinal cohort community study in DRC. Kwashiorkor was a major cause of death and these children have not been taken out of the analysis, although all kwashiorkor deaths occurred in children over 18 months of age, 28% of the deaths occurred in infants <6months of age and these have been included in all analyses; this makes derivation of the relationship between MUAC and WHZ in the 6-60 month old children problematic from the reported data. Measles and diarrhoea were “uncommon”, but malaria was a common cause of death. Interestingly, the incidence of kwashiorkor is about 16 times that of marasmus. If the duration of marasmus is 90 days then the duration of an episode of kwashiorkor is around 7 days. This is why oedematous children are not usually found in surveys, but frequently present to hospital, and why estimates of disease burden based on prevalence rather than incidence are misleading in terms of deriving public health policy.

**References**

1. Aguayo VM, Aneja S, Badgaiyan N, Singh K. Mid upper-arm circumference is an effective tool to identify infants and young children with severe acute malnutrition in India. Public health nutrition 2015, 18: 3244-3248. <https://www.cambridge.org/core/services/aop-cambridge-core/content/view/S1368980015000543>
2. Grellety E, Shepherd S, Roederer T, Manzo ML, Doyon S, Ategbo EA et al. Effect of mass supplementation with ready-to-use supplementary food during an anticipated nutritional emergency. PLoS ONE 2012, 7: e44549. <http://www.ncbi.nlm.nih.gov/pmc/articles/PMC3440398/pdf/pone.0044549.pdf>
3. Grellety E, Krause LK, Shams EM, Porten K, Isanaka S. Comparison of weight-for-height and mid-upper arm circumference (MUAC) in a therapeutic feeding programme in South Sudan: is MUAC alone a sufficient criterion for admission of children at high risk of mortality? Public Health Nutrition 2015, 18: 2575-2581. <http://www.epicentre.msf.org/sites/preprod.epicentre.actency.fr/files/661_Comparison%20of%20weight-for-height.pdf>
4. Isanaka S, Guesdon B, Labar AS, Hanson K, Langendorf C, Grais RF. Comparison of Clinical Characteristics and Treatment Outcomes of Children Selected for Treatment of Severe Acute Malnutrition Using Mid Upper Arm Circumference and/or Weight-for-Height Z-Score. PLoS One 2015, 10: e0137606. <http://journals.plos.org/plosone/article?id=10.1371/journal.pone.0137606>
5. Lowlaavar N, Larson CP, Kumbakumba E, Zhou G, Ansermino JM, Singer J et al. Pediatric in-hospital death from infectious disease in Uganda: Derivation of clinical prediction models. PloS one 2016, 11: e0150683. <http://journals.plos.org/plosone/article?id=10.1371/journal.pone.0150683>
6. LaCourse S, Chester FM, Preidis G, McCrary LM, Maliwichi M, McCollum ED et al. Lay-screeners and use of WHO growth standards increase case finding of hospitalized Malawian children with severe acute malnutrition. Journal of tropical pediatrics 2014, 61: 44-53. DOI: 10.1093/tropej/fmu065
7. Olofin I, Guesdon B, Roberfroid D. Associations of suboptimal MUAC and WHZ combinations with child mortality: a pooled analysis. Action Against Hunger, Research for Nutrition Conference 2016. <http://files.ennonline.net/attachments/2604/ACF-conference-special-section.pdf>
8. Berkley J, Mwangi I, Griffiths K, Ahmed I, Mithwani S, English M et al. Assessment of severe malnutrition among hospitalized children in rural Kenya: comparison of weight for height and mid upper arm circumference. Jama 2005, 294: 591-597. <http://jamanetwork.com/journals/jama/fullarticle/201328>
9. Chiabi A, Mbanga C, Mah E, Nguefack DF, Nguefack S, Fru F et al. Weight-for-Height Z Score and Mid-Upper Arm Circumference as Predictors of Mortality in Children with Severe Acute Malnutrition. Journal of tropical pediatrics 2017. <https://academic.oup.com/tropej/article-abstract/63/4/260/2671088>
10. Golden MH. Comment on WHZ and MUAC for diagnosis of Severe malnutrition by Chiabi A et al. Journal of tropical pediatrics 2017, 0: 1-2. DOI: 10.1093/tropej/fmx008
11. Sachdeva S, Dewan P, Shah D, Malhotra RK, Gupta P. Mid-upper arm circumference v. weight-for-height Z-score for predicting mortality in hospitalized children under 5 years of age. Public Health Nutrition 2016, 1-8. <https://www.cambridge.org/core/services/aop-cambridge-core/content/view/S1368980016000719>
12. Briend A, Mwangome MK, Berkley JA. Using Mid-Upper Arm Circumference to Detect High-Risk Malnourished Patients in Need of Treatment. In: Preedy V, Patel V. (eds) Handbook of Famine, Starvation, and Nutrient Deprivation. Springer, Cham 2017. DOI: 10.1007/978-3-319-40007-5_11-1
13. Grant A, Njiru J, Okoth E, Awino I, Briend A, Murage S, Abdirahman S, Myatt M. Comparing performance of mothers using simplified mid-upper arm circumference (MUAC) classification devices with an improved MUAC insertion tape in Isiolo County, Kenya. Arch Public Health. 2018;76(1). <https://doi.org/10.1186/s13690-018-0260-x>
14. Heikens GT, Manary MJ, Trehan I. African children with severe pneumonia remain at high risk for death even after discharge. Paediatr Perinat Epidemiol 2017; 31(3): 243-244. <https://doi.org/10.1111/ppe.12350>
15. Burza S, Mahajan R, Marino E, Sunyoto T, Shandilya C, Tabrez M et al. Seasonal effect and long-term nutritional status following exit from a Community-Based Management of Severe Acute Malnutrition program in Bihar, India. Eur J Clin Nutr 2015. <http://www.nature.com/ejcn/journal/v70/n4/full/ejcn2015140a.html>
16. Schwinger C, Lunde TM, Andersen P, Kismul H, Van den Broeck J. Seasonal and spatial factors related to longitudinal patterns of child growth Bwamanda, DR Congo. Earth Perspectives. 2014;1:26. <https://doi.org/10.1186/s40322-014-0026-8>
17. Mogeni P, Twahir H, Bandika V, Mwalekwa L, Thitiri J, Ngari M et al. Diagnostic performance of visible severe wasting for identifying severe acute malnutrition in children admitted to hospital in Kenya. Bull WHO. 2011, 89: 900-906. <http://www.who.int/bulletin/volumes/89/12/11-091280/en/>
18. Sylla A, Gueye M, Keita Y, Seck N, Seck A, Mbow F et al. Dehydration and malnutrition as two independent risk factors of death in a Senegalese pediatric hospital. Archives de pediatrie: organe officiel de la Societe francaise de pediatrie 2015, 22: 235-240. DOI: 10.1016/j.arcped.2014.11.024
19. Vella V. An epidemiological analysis of the determinants of childhood malnutrition and mortality in southwest Uganda. PhD thesis London School of Hygiene & Tropical Medicine; 1990. <http://researchonline.lshtm.ac.uk/682267/1/296416.pdf>
20. Vella V, Tomkins A, Borghesi A, Migliori GB, Ndiku J, Adriko BC. Anthropometry and childhood mortality in northwest and southwest Uganda. Am J Public Health. 1993;83(11):1616–8. DOI: 10.2105/AJPH.83.11.1616
21. Vella V, Tomkins A, Ndiku J, Marshal T, Cortinovis I. Anthropometry as a predictor for mortality among Ugandan children, allowing for socio-economic variables. Eur J Clin Nutr. 1994;48(3):189–97.
22. Dramaix M, Hennart P, Brasseur D, Bahwere P, Mudjene O, Tonglet R et al. Serum albumin concentration, arm circumference, and oedema and subsequent risk of dying in children in central Africa. Bmj 1993, 307: 710-713. DOI: https://doi.org/10.1136/bmj.307.6906.710
23. Girum T, Kote M, Tariku B, Bekele H. Survival status and predictors of mortality among severely acute malnourished children< 5 years of age admitted to stabilization centers in Gedeo Zone: a retrospective cohort study. Therapeutics and Clinical Risk Management 2017, 13: 101. <https://doi.org/10.2147/TCRM.S119826>
24. Savadogo L, Zoetaba I, Donnen P, Hennart P, Sondo BK, Dramaix M. Management of severe acute malnutrition in an urban nutritional rehabilitation center in Burkina Faso. Revue d'epidemiologie et de sante publique 2007, 55: 265-274. DOI: 10.1016/j.respe.2007.05.006
25. Garenne M, Maire B, Fontaine O, Dieng K, Briend A. Risques de décès associés à différents états nutritionnels chez l'enfant d'âge préscolaire: étude réalisée à Niakhar (Sénégal), 1983-1986. ORSTOM Dakar; 1987. <http://horizon.documentation.ird.fr/exl-doc/pleins_textes/divers11-10/24687.pdf>
26. Garenne M, Willie D, Maire B, Fontaine O, Eeckels R, Briend A et al. Incidence and duration of severe wasting in two African populations. Public health nutrition 2009, 12: 1974. <https://www.cambridge.org/core/services/aop-cambridge-core/content/view/S1368980009004972>
27. Van Den Broeck J, Eeckels R, Vuylsteke J. Influence of nutritional status on child mortality in rural Zaire. Lancet 1993, 341: 1491-1495. <http://www.sciencedirect.com/science/article/pii/014067369390632Q>
