# Supplementary figures and images for: Severely malnourished children with a low weight-for-height have similar mortality to those with a low mid-upper-arm-circumference: II. Systematic literature review and meta-analysis
Source: Nutr J. 2018 Sep 15;17:80. doi: 10.1186/s12937-018-0383-5 (PMC6138903; doi:10.1186/s12937-018-0383-5)

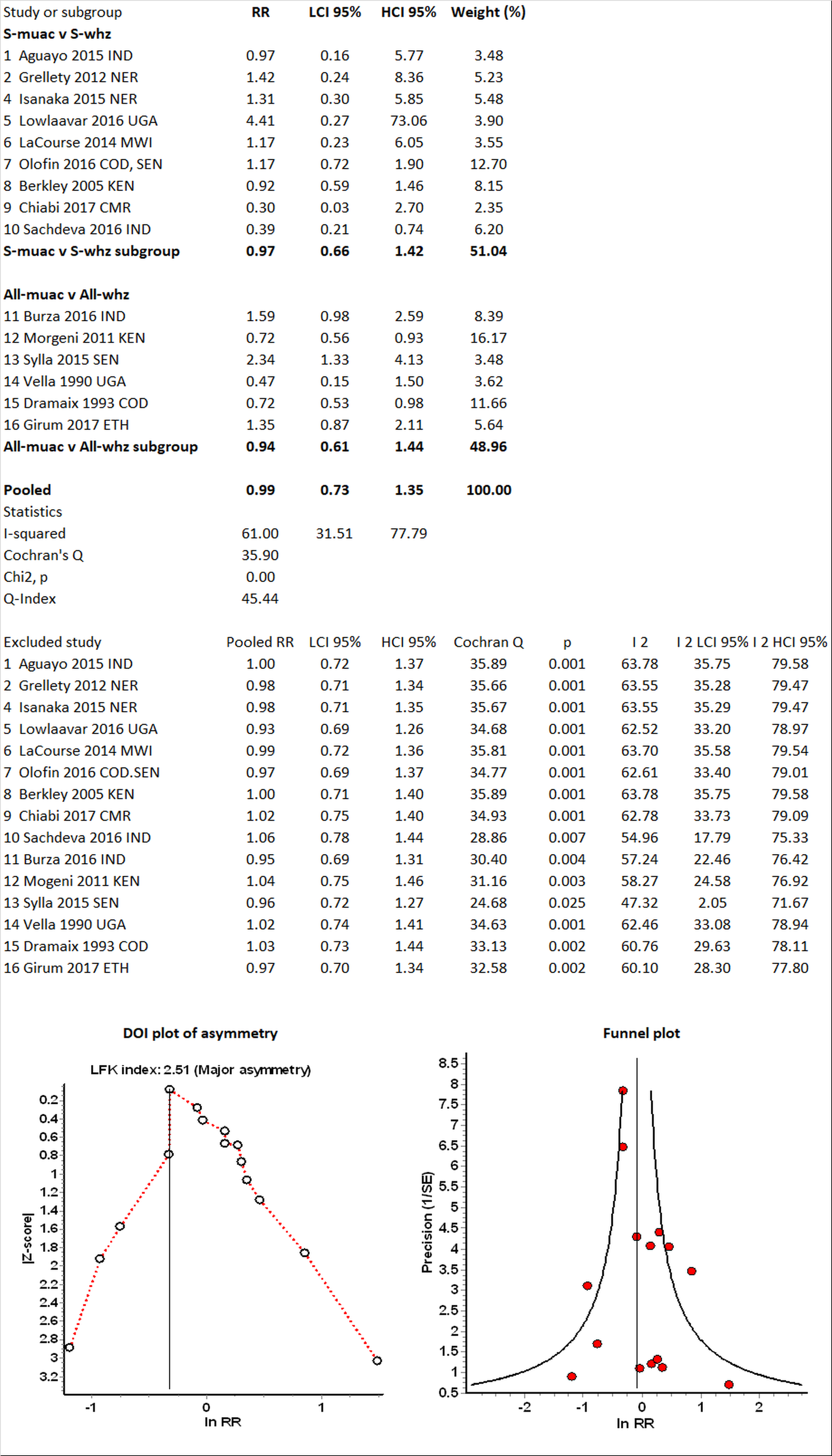

Supplement: Supplementary file 3 — Figure S1. Statistics, Sensitivity analysis, DOI plot and funnel plot corresponding to Fig. 6. (TIF 2826 kb) [file 12937_2018_383_MOESM3_ESM.tif]
